# Transcriptomic analysis reveals candidate genes regulating development and host interactions of *Colletotrichum fructicola*

**DOI:** 10.1186/s12864-018-4934-0

**Published:** 2018-07-28

**Authors:** Xiaofei Liang, Shengping Shang, Qiuyue Dong, Bo Wang, Rong Zhang, Mark L. Gleason, Guangyu Sun

**Affiliations:** 1 State Key Laboratory of Crop Stress Biology in Arid Areas and College of Plant Protection, Northwest A&F University, Yangling, 712100 Shaanxi Province China; 2 Department of Plant Pathology and Microbiology, Iowa State University, Ames, IA 50011 USA

**Keywords:** Glomerella, Leaf spot, GLS, Colletotrichum fructicola, Conidium, Triacylglycerol, Lipid, Appressorium

## Abstract

**Background:**

*Colletotrichum* is a fungal genus in Ascomycota that contain many plant pathogens. Among all *Colletotrichum* genomes that have been sequenced, *C. fructicola* contains the largest number of candidate virulence factors, such as plant cell wall degrading enzymes, secondary metabolite (SM) biosynthetic enzymes, secreted proteinases, and small secreted proteins. Systematic analysis of the expressional patterns of these factors would be an important step toward identifying key virulence determinants.

**Results:**

In this study, we obtained and compared the global transcriptome profiles of four types of infection-related structures: conidia, appressoria, infected apple leaves, and cellophane infectious hyphae (bulbous hyphae spreading inside cellophane) of *C. fructicola*. We also compared the expression changes of candidate virulence factors among these structures in a systematic manner. A total of 3189 genes were differentially expressed in at least one pairwise comparison. Genes showing *in planta*-specific expressional upregulations were enriched with small secreted proteins (SSPs), cytochrome P450s, carbohydrate-active enzymes (CAZYs) and secondary metabolite (SM) synthetases, and included homologs of several known candidate effectors and one SM gene cluster specific to the *Colletotrichum* genus. In conidia, tens of genes functioning in triacylglycerol biosynthesis showed coordinately expressional upregulation, supporting the viewpoint that *C. fructicola* builds up lipid droplets as energy reserves. Several phosphate starvation responsive genes were coordinately up-regulated during early plant colonization, indicating a phosphate-limited *in planta* environment immediately faced by biotrophic infectious hyphae.

**Conclusion:**

This study systematically analyzes the expression patterns of candidate virulence genes, and reveals biological activities related to the development of several infection-related structures of *C. fructicola*. Our findings lay a foundation for further dissecting infection mechanisms in *Colletotrichum* and identifying disease control targets.

**Electronic supplementary material:**

The online version of this article (10.1186/s12864-018-4934-0) contains supplementary material, which is available to authorized users.

## Background

The *Colletotrichum gloeosporioides* species complex (CGSC) encompasses over 20 fungal species in Ascomycota that are morphologically indistinguishable, but can be differentiated with the aid of a common set of DNA markers [1–3]. *C. fructicola* Prihastuti, Cai & Hyde is a CGSC species that was first established based on isolates from coffee (*Coffea arabica*) berries and is synonymous with *C. ignotum* and *Glomerella cingulata* var. *minor* [3–5]. It has a broad host range and is cosmopolitan, having been identified on over 50 plant species throughout Asia, Africa, Europe, America, and Australia [6]. The wide occurrence of certain *C. fructicola* diseases, such as Glomerella leaf spot of apple and Camellia anthracnose, has resulted in large economic losses for growers of these commodities [2, 7].

*Colletotrichum* pathogens are hemibiotrophs with a multistage infection process. Attached conidia germinate and differentiate dome-shaped appressoria on plant surfaces, underneath which penetration pegs form and penetrate epidermal cells. The pathogen then differentiates bulbous biotrophic hyphae which are enveloped by an intact host plasma membrane; biotrophic hyphae spread across living host cells before differentiating thin necrotrophic hyphae that kill and destroy host tissues [8, 9]. Genomic and transcriptomic analyses have revealed a range of candidate factors contributing to *Colletotrichum* virulence at biotrophic and/or necrotrophic phases [10–12]. Biotrophic factors, such as candidate effectors and secondary metabolite (SM) biosynthetic enzymes, generally express early in the infection process and function in host defense suppression or cellular activity manipulation; necrotrophic factors, on the other hand, generally express late in the infection process and function in necrosis induction or macromolecule hydrolysis [8, 11, 12]. Among candidate virulence factors, SM enzymes and candidate effectors are the best characterized [8, 13–15]. Deletion of the *C. graminicola* Sfp-type 4′-phosphopantetheinyl transferase gene, which functions in activating the fungal SM enzymes polyketide synthase (PKS) and nonribosomal peptide synthetase (NRPS), renders these mutants nonpathogenic [16]. Candidate effectors showing defense-suppressive, necrosis-inducing, transcription-regulating, and carbohydrate-binding activities have also been identified [14, 17–20]. Genes coding for these effectors, however, collectively represent only a small portion of the rich repertoire of *Colletotrichum* candidate virulence factors uncovered by genome sequencing.

Among known *Colletotrichum* genomes, *C. fructicola* encodes the largest number of putative plant cell wall degrading enzymes (PCWDEs), secreted proteases, candidate effectors and SM enzymes [11]. Functional characterization of these genes would be a critical step toward understanding the infection mechanisms of *C. fructicola*. Given the long candidate gene list, it is necessary to prioritize genes and associated transcriptomes to save time and resources. In this study, the global transcriptomic profiles of four types of infection-related structures (conidia, appressoria, cellophane infectious hyphae, and infected plant tissue) were determined and compared. The objectives were to analyze the global expression patterns of known candidate virulence genes and identify candidate genes regulating fungal infection-related developments and host interactions.

## Methods

### Fungal isolate and plant materials

The *C. fructicola* isolate SQ06 selected for RNA sequencing was isolated from a Glomerella leaf spot (GLS) lesion on apple (cv. Gala) in Shangqiu city, Henan Province, China. The isolate was cultured on potato dextrose agar (PDA) and preserved as a glycerol stock (15%) at − 80 °C in the Fungal Laboratory, College of Plant Protection, Northwest A&F University. Trees of apple cv. Gala, which is highly susceptible to GLS, were grown in an orchard near Yangling, Shaanxi Province, China.

### Sample preparation

*Conidia (CON)*: conidial production was induced by culturing in potato dextrose broth (PDB) (25 °C, shaken at 150 rpm [revolutions per minute], under natural light) as previously described [21]. Six 100 mL Erlenmeyer flasks, each containing 50 mL PDB medium, were inoculated with mycelial plugs (four plugs in each flask). After being cultured for five days, conidia were harvested by filtering through three layers of Miracloth (Merck Millipore, catalog number: 475855) to remove mycelia, centrifuging for 5 min at 12,000 rpm and 4 °C, discarding the supernatant, and washing the pellet twice with sterile water followed each time by centrifugation. Purified conidial pellets were immediately frozen in liquid nitrogen and stored at − 80 °C until usage. *Appressoria (APP)*: appressorium formation was induced by spraying 200 μL of conidial suspension (1 × 10^7^/mL) on a 9-mm-diameter layer of cellophane placed on top of 2% water agar, and incubated at 25 °C. At 8 h post inoculation (hpi), cellophane sheets bearing appressoria and germ tubes were cut into small pieces, immediately frozen in liquid nitrogen and stored at − 80 °C until usage. *Cellophane infectious hyphae (CIH)*: 200 μL of a conidial suspension (1 × 10^7^/mL) was sprayed on a 9-cm-diameter circle of cellophane placed on top of 2% water agar amended with 0.01% yeast extract, which was incubated at 25 °C until tissue harvest. Germinated conidia formed vegetative hyphae on the membrane surface, while at the same time penetrated cellophane and formed CIH. At 36 hpi, conidia and vegetative hyphae on the cellophane surface were removed by gentle fingertip rubbing and rinsing with tap water. The CIH were then harvested in the same way as described for harvesting APP. *Infected Leaf (IL)*: apple (cv. Gala) leaves were harvested from trees growing in the Yangling orchard, then brought back to the lab. Fully expanded young leaves (harvested in August 2015) were surface-rinsed with tap water, sterilized by rubbing with alcohol cotton ball, air-dried, placed in moist chamber and immediately used for inoculation, for which a conidial suspension (5 × 10^6^/mL in 0.05% tween 20) was brushed onto the adaxial leaf surface, then incubated in a moist chamber at room temperature (~ 25 °C). At 48 hpi, five random leaves bearing uniform tiny lesions were selected, leaf veins were cut away, and the remaining leaf tissues were mixed and immediately frozen in liquid nitrogen, then stored at − 80 °C for later RNA extraction.

### RNA isolation and sequencing

For each sample type, three independent samples were collected for subsequent RNA extraction and sequencing. Total RNAs were extracted with a TIANGEN RNAsimple Kit (TIANGEN, DP419, Beijing, China). RNA degradation and contamination were monitored on 1% agarose gels. RNA concentration was measured using Qubit® RNA Assay Kit in Qubit® 2.0 Fluorometer (Life Technologies, CA, USA), and RNA integrity was assessed using the RNA Nano 6000 Assay Kit of the Agilent Bioanalyzer 2100 system (Agilent Technologies, CA, USA).

A total of 1.5 μg RNA per sample was used as input material for RNA sequencing. Library preparation and library sequencing were performed at the Novogene Bioinformatics Institute (Beijing, China). Sequencing libraries were prepared using NEB Next® Ultra™ RNA Library Prep Kit for Illumina® (NEB, MA, USA), and were then sequenced on an Illumina Hiseq 4000 platform with a 150 bp pair-ended strategy. The intended sequencing depths for CON, APP, and CIH were 1 Gb clean bases per library, and the intended sequencing depth for IL was 6 Gb per library to raise the fungal reads.

### Data analysis

For quality control, error-prone reads and reads containing adapter sequences were identified and the corresponding read-pairs were removed through in-house perl scripts developed by the Novogene Bioinformatics Institute (Beijing, China). Based on literature recommendation [22], low-quality reads were defined based on the criterion that the number of N bases exceeded 10% of the total read length, or that the number of error-prone bases (quality score < =5) exceeded 50% of the total read length. After filtration, the ratio of accurate bases (Phred score > 20) ranged from 95.17 to 97.37% among different libraries, and the ratio of highly accurate bases (Phred score > 30) ranged from 89.53 to 93.31%.

Bowtie 2 version 2.2.6 [23] and TopHat version 2.1.0 [24] were used to align clean reads to the *C. fructicola* 1104–7 reference genome (GenBank accession no. MVNS00000000). For reads mapping, a maximum of two mismatches and two gaps were allowed, concordant mapping to the same genome location was enforced, and reads with multiple hits were not allowed. Reads mapped to each predicted gene model (17,827 in total) were counted in HTSeq version 0.6.0 [25]. Saturation analyses of library sequencing depths were performed with the NOISeq R package [26]. The inter-replicate CPM (counts per million) variability of individual genes were then examined to estimate the CPM filtering threshold above which precise gene expression estimation could be achieved. Subsequently, DESeq2 [27] and edgeR [28] analyses were performed to identify differently expressed genes (DEGs). In DESeq2 and edgeR, differential expressions were tested with a fold-change threshold, and the lfc (log fold change) parameters were both set to 3, equivalent to 8-fold expressional difference. In DESeq2, the filtering alpha value was set to 0.05, and the filtering parameter in edgeR was ‘CPM ≥ 5 in at least three libraries’. For each pairwise combination, only DEGs identified by both DESeq2 and edgeR were kept; these genes were further filtered to ensure that the average CPM of the up-regulated sample was larger than a predetermined CPM filtering threshold (5 for CON, APP and CIH; 50 for IL). The DEG identification procedure was shown in Additional file 1: Figure S1 and the complete bioinformatic command lines were reported in Additional file 1: Table S1.

Identification of candidate virulence genes from *C. fructicola*, including putative cytochrome P450s, transporters, small secreted proteins (SSPs), carbohydrate-active enzymes (CAZYs), secondary metabolite (SM) synthetases, and secretory proteases from the *C. fructicola* 1104–7 genome has been described previously [29]. Additional genes of interest were identified by BLAST search or local Interproscan database search [30]. CPMs were used for hierarchical clustering analysis and for heatmap generation. Hierarchical clustering was performed with GeneCluster version 3.0 [31]. CPM values were added with a prior value of 0.125, log-transformed and centered with means prior to hierarchical clustering with the average link method. Cluster outcome was visualized with Java TreeView version 1.1.6r4 [32]. Functional enrichment tests of PFAM domains and gene ontologies (GOs) were performed with FunRich version 2.1.2 [33], in which hypergeometric tests were performed and *P*-values were adjusted with the Benjamini-Hochberg procedure to obtain false discovery rates (FDRs).

### Expressional validation by qRT-PCR of conidium-related genes

To determine whether ‘conidium-related’ genes identified by RNA-seq were stage-specific (showing rapid expressional down-regulation upon conidial germination), we extracted total RNAs from conidia harvested from 5-day-old PDB culture (150 rpm, 25 °C), conidia germinated in liquid shake PDB (1 × 10^4^ conidia/mL, 200 rpm, 25 °C, 12 h), and conidia germinated on cellophane laid on top of PDA (2 × 10^5^ conidia/plate, 25 °C, 12 h). RNAs were also extracted from appressoria (1 × 10^6^ conidia/mL, drop-inoculated on onion epidermis, 8 h). We used onion epidermis to induce appressorium formation because onion epidermis has a higher inducing efficiency and resembles leaf surface more closely than cellophane membrane. RNAs were also extracted from infected apple leaves (cv. Gala) at different times after inoculation (24, 36, 48, 60, and 72 h). RNAs were extracted with a TIANGEN RNAsimple Kit (catalog number: DP419), and contaminated DNAs were removed by in-column DNase treatment. Purified RNAs were reverse-transcribed into cDNAs with a cDNA synthesis kit (Thermo Scientific, catalog number: K1621). SYBR Green based qRT-PCR amplifications were performed with a StepOnePlus™ Real-Time PCR System, and PCRs were performed with the 2 × RealStar Green Fast Mixture (GenStar, catalog number: A301) using the following program: 5 min at 94 °C, followed by 40 cycles of 94 °C for 10 s and 60 °C for 30 s. The alpha-tubulin (TUB) gene was chosen as the internal control and relative quantifications were calculated based on the delta delta Ct approach [34]. For statistical analysis, relative gene expression fold changes were compared using one-way permutation test with the *coin* and *rcompanion* R packages [35]. qRT-PCR primers used in this study are listed in Additional file 1: Table S2.

## Results

### Tissues sampled for RNA sequencing

**Fig. 1:**
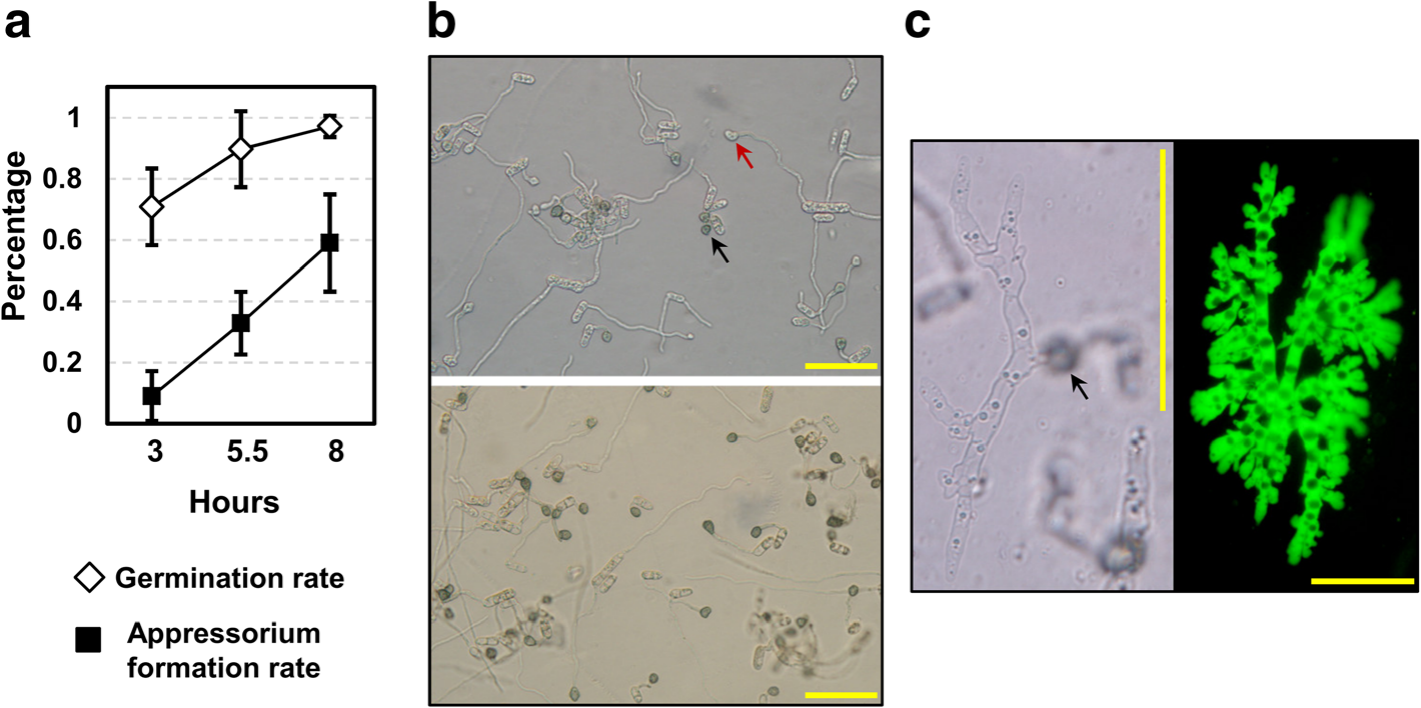
Development of *C. fructicola* appressoria and cellophane infectious hyphae. **a** Conidial germination and appressorium formation on cellophane surface. Plotted means and standard deviations were based on counts from 10 randomly chosen optical fields, each with around 35 conidia. Appressorium formation rate was calculated on per-germ-tube basis. **b** Top, at 8 h post inoculation (hpi), appressoria at ends of short germ tubes were melanized (black arrow) whereas those at ends of long germ tubes were still hyaline (red arrow). Bottom, all appressoria became heavily melanized by 10.5 hpi. **c** Cellophane infectious hyphae. Left, nascent infectious hyphae formed at an appressorium penetration site (arrowhead) at 18 hpi; Right, bulbous cellophane infectious hyphae observed with a GFP-labeled strain at 36 hpi. Scale bars in **b** and **c** represent 50 μm

**Fig. 2:**
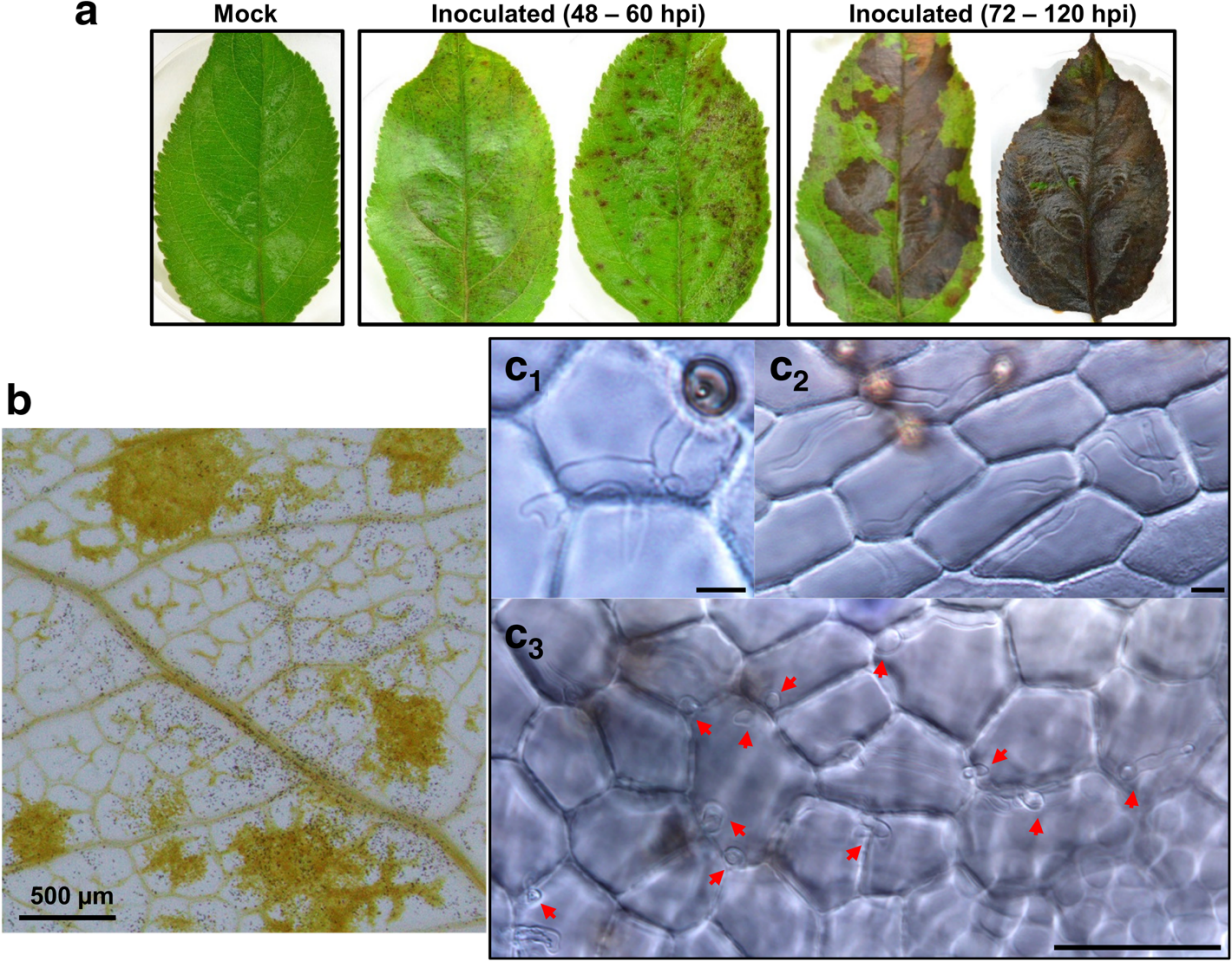
Infection characteristics of *C. fructicola* on detached apple leaf. **a**. Typical symptom development of detached leaf inoculated with conidial suspension (5 × 10^6^/mL in 0.05% Tween 20). **b**. At 48 hpi, appressoria formed abundantly on leaf surface (tiny black spots) whereas necrotic lesions formed sporadically (yellow color). **c**. At 54 hpi, infectious hyphae spread both in the epidermal layer (**c**_**1**_, **c**_**2**_) and downward to the mesophyll (**c**_**3,**_ red arrows). Scale bars in **c** represent 20 μm

Liquid shake PDB culture efficiently induced conidial formation; the concentration exceeded 10^7^ per mL at the collection time (5 dpi). On the cellophane inductive surface, approximately 70% of conidia germinated at 3 hpi and the germination rate reached ~ 97% at 8 hpi (Fig. 1a). Appressorium formation incidence (based on germinated conidia) increased from ~ 10% at 3 hpi to ~ 60% at 8 hpi (Fig. 1a). Appressoria produced from short germ tubes melanized earlier than those produced from long germ tubes (Fig. 1b). By 10.5 hpi, most appressoria had become heavily melanized. Appressorial tissue was harvested at 8 hpi, when the appressorium formation frequency was high and appressoria at various developmental stages were present. On cellophane, *C. fructicola* appressoria penetrated and formed cellophane infectious hyphae (CIH) from 18 hpi onward, which were thick, bulbous, heavily branched and tightly aligned, resembling the biotrophic infectious hyphae formed during *in-planta* colonization (Fig. 1c). On inoculated apple leaves, *C. fructicola* conidia differentiated abundant appressoria; necrotic lesions became visible and rapidly merged together from 48 hpi onward (Fig. 2a, b). Inside the lesions, infectious hyphae spread across epidermal cells and into mesophylls (Fig. 2c).

### Quality control and identification of differentially expressed genes

A total of 146.5 million clean RNA-Seq reads, representing 44 Gb nucleotide bases, were generated. Of these, 36.7 million reads could be unequivocally aligned to the 1104–7 reference genome (concordant unique pairs, see details in Additional file 1: Table S3). Despite the IL libraries had over 6-fold more sequence reads, the numbers of mapped reads were much fewer compared with those of CON, APP, and CIH, the average alignment rates were 75.4, 75.4, 73.7 and 0.81%; and the average number of aligned reads were 4.75, 3.68, 3.55, and 0.27 million. NOISeq simulation predicted that one million new reads would detect 190–370 new genes (counts ≥5) for CON, APP, and CIH libraries; but would detect 4018–7576 new genes (counts ≥5) for the IL libraries (Additional file 1: Figure S2). Thus, in terms of new gene detection, the CON, APP and CIH libraries were well-saturated whereas the IL libraries were not.

We next examined the inter-replicate CPM variability, so as to estimate the CPM filtering threshold above which precise gene expression estimation (e.g. CPM) could be achieved. For each gene, its ‘relative CPM error value’ was calculated as the relative difference between its CPM in one replicate (chosen arbitrarily) and the average CPM calculated from the rest two replicates. In theory, the relative inter-replicate CPM error would be stationary for highly expressed genes whereas unsaturated genes would have larger relative CPM errors due to Poisson noise interference. By removing low-expression genes, the genome-wide relative CPM errors should gradually stabilize, enabling the optimal CPM filtering thresholds to be determined. The distributions of genome-wide relative CPM errors are plotted in Additional file 1: Figure S3a. For CON, APP, and CIH, removing genes with CPM < 5 controlled the relative CPM errors to within 0.6 for 99.3, 96.7, and 97.4% of genes respectively, and the error distributions tended to stabilize when more stringent cutoff values were applied. For the IL sample, a CPM cutoff of 50 controlled the relative CPM errors to within 1.0 for 96.0% of genes, and the error distributions also stabilized thereafter. Based on these results, we determined the optimal CPM filtering threshold for CON, APP, and CIH to be 5, and the optimal threshold for IL to be 50. Additional file 1: Figure S3b shows the correlation plots before and after applying these filtering thresholds in CON, APP, CIH and IL. Additional file 1: Figure S4 shows the density plot distribution of relative CPM errors for CIH and IL.

The 12 RNA-seq libraries formed sample type-defined clusters in pairwise distance heatmap (Additional file 1: Figure S5a), replicates neighbored together whereas different biological conditions were separated. Based on a set of arbitrary cutoffs (CPM ≥ 1 in all three libraries for CON, APP, and CIH; CPM ≥ 10 in all three libraries for IL), 12,511 out of 17,827 predicted gene models were detected to be expressed, which included 9027, 10,489, 10,740, and 7117 genes in CON, APP, CIH and IL respectively (Additional file 1: Figure S5b).

DESeq2 and edgeR identified 3555 and 5326 differently expressed genes (DEGs), respectively, in at least one of the six pairwise comparisons (Additional file 1: Figure S5c). Up to 3433 DEGs were supported by both programs. Further CPM filtering (average CPM ≥ 5 in up-regulated condition if being CON, APP, or CIH, and average CPM ≥ 50 in up-regulated condition if being IL) removed 244 genes, producing 3189 final DEGs (Additional file 1: Figure S5c). These DEGs showed at least 8-fold expressional difference, were supported by both programs, and the CPMs for the up-regulated conditions were above the CPM thresholds, and thus were highly likely to be true DEGs. Additional file 1: Figure S6a plotted the gene average CPM distribution among all six pairwise comparisons; the average CPMs of DEGs were clearly more variable among samples compared with non-DEGs. Additional file 1: Figure S6b plotted the proportion of genes which had CPM values less than a given threshold in at least one of the two compared samples. Except for CPM 1, the corresponding proportions were always higher for DEGs than for non-DEGs and the genome background (all genes considered). This pattern supported the interpretation that the DEG identification procedure enriched genes being lowly-expressed at one infection stage but highly activated at another stage.

To validate the gene expression profile obtained with RNA-Seq, the expression of 28 genes was further quantified using qRT-PCR. Fourteen chosen genes were related to triacylglycerol biosynthesis and conidial development, their relative CON-to-APP fold change values were calculated with both qRT-PCR and RNA-Seq, which were closely correlated (Pearson correlation coefficient 0.82). Another 14 chosen genes were functionally related to phosphate starvation response and quinate metabolism; their relative IL(48 hpi)-to-APP fold change values were also compared, and the Pearson correlation coefficient between RNA-seq and qRT-PCR was 0.81. These results suggest that the transcriptome analysis performed in this study is reliable.

**Fig. 3:**
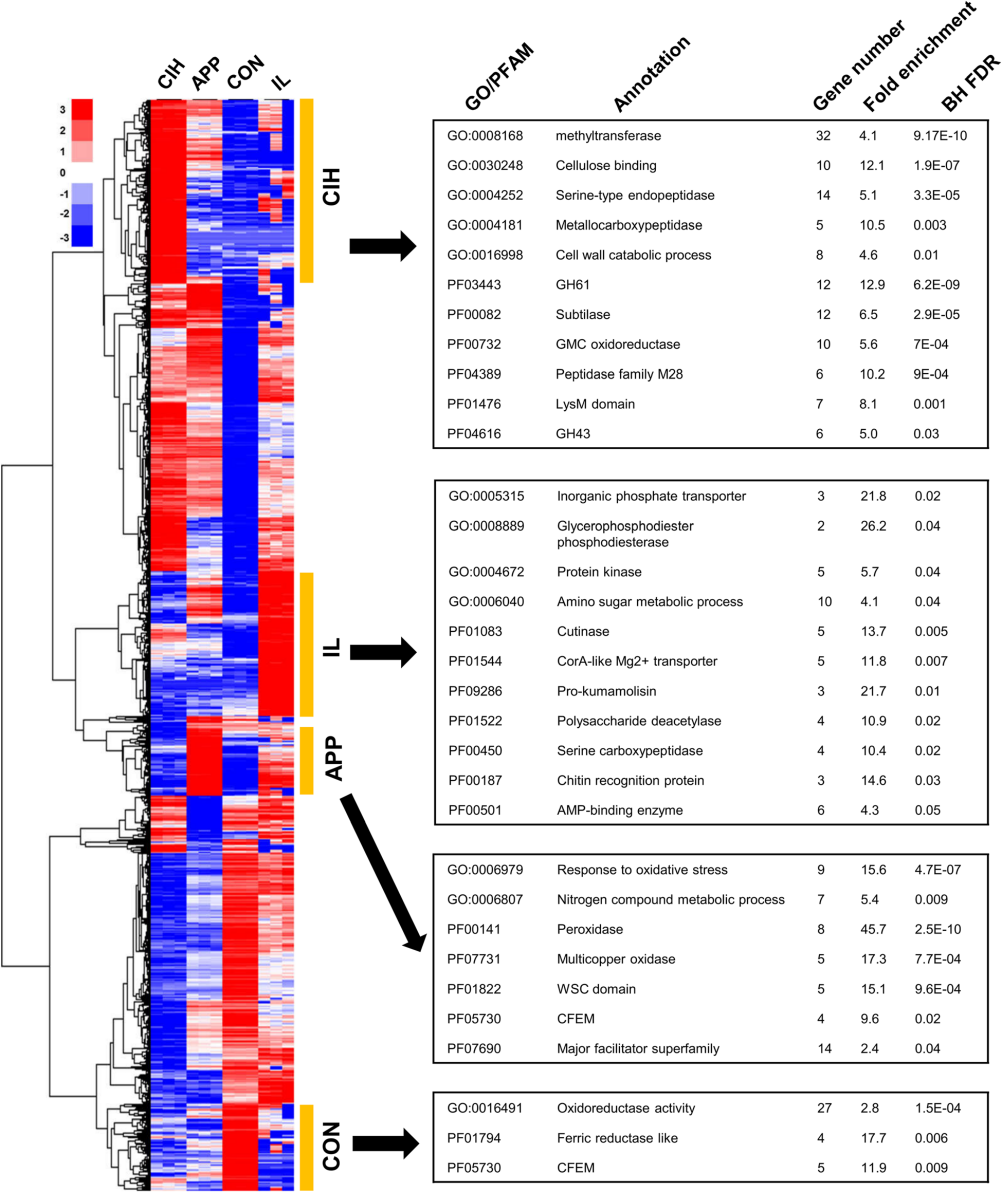
Differently-expressed genes identified in this study. Left, hierarchical clustering heatmap. Levels of expression are represented as moderated log2 ratios of CPMs. Right, significantly enriched gene ontology (GO) terms and PFAM domains for the four structure-specific clusters defined in the heatmap. Hypergeometric enrichment tests were performed against the genome background, and BH FDR represents Benjamini and Hochberg’s adjusted *P*-value

Hierarchical clustering analysis was performed with DEGs based on their CPM profile among samples, this analysis identified four gene clusters made up of genes being strongly up-regulated in CON, IL, APP or CIH compared with the other three tissue types (Fig. 3 left). PFAM domains and gene ontology (GO) terms being significantly enriched for individual clusters are shown in Fig. 3 right. The annotation and pairwise fold changes of individual DEGs are shown in Additional file 2: Table S4.

### Global expression patterns of candidate virulence factors

**Fig. 4:**
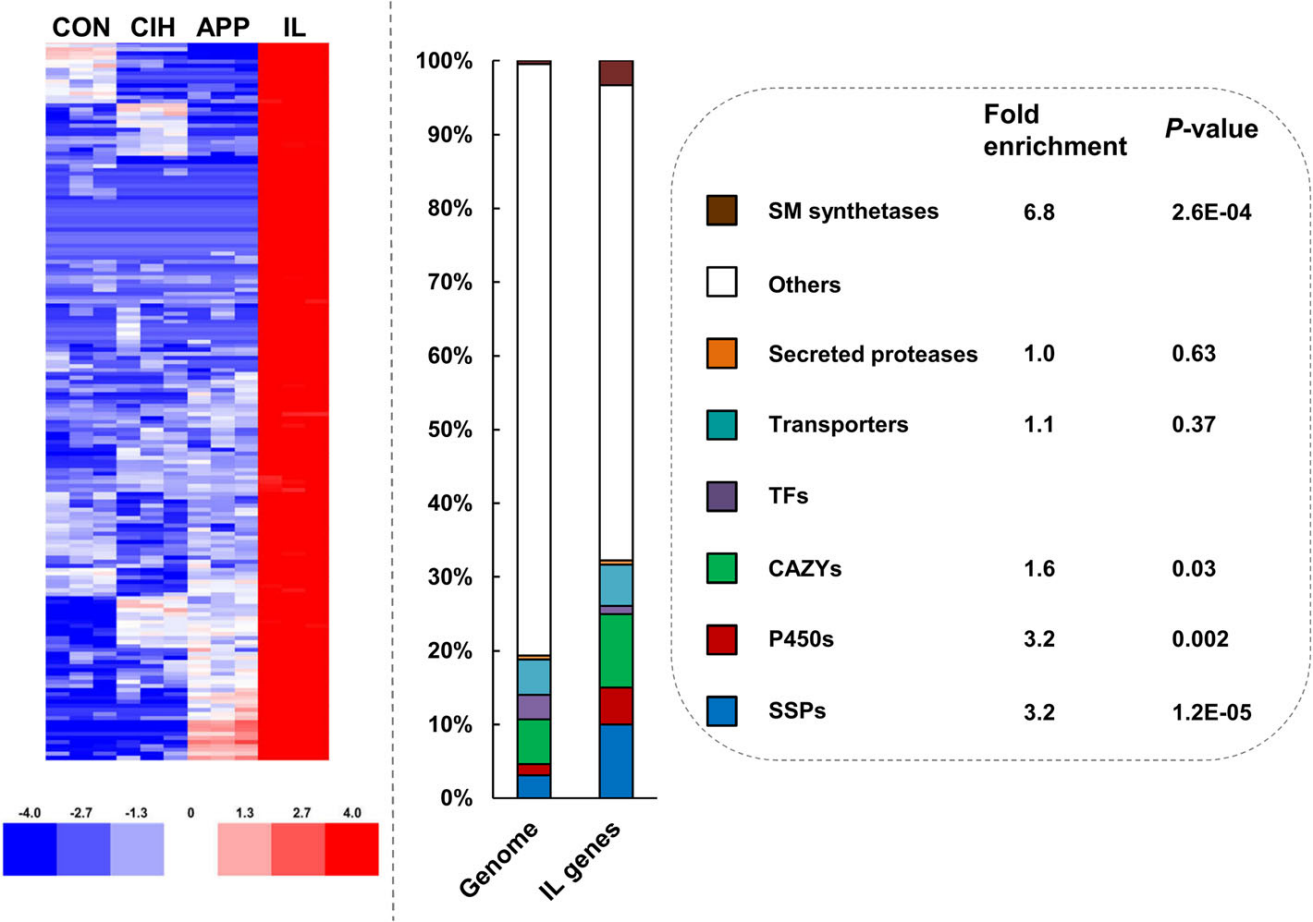
Genes showing *in planta*-specific expression (IL genes) are enriched with candidate virulence factors. Left, log2 ratios of CPMs. Right, functional enrichment analysis, *P-values* are calculated based on hypergeometric tests

The *C. fructicola* 1104–7 genome contained 88 secondary metabolite (SM) synthetases and 52 SM clusters (predicted by SMURF), 552 small secreted proteins (SSPs, containing predicted secretory signals, lacking predicted transmembrane region, amino acid length < 300), 1129 CAZYs, and 96 secretory proteases. Among 180 genes showing *in planta*-specific upregulations (Log_2_^Fold Change^ ≥ 3 compared with each of the other three conditions), SSPs, P450s, CAZYs and SM synthetases were significantly enriched compared to the genome background (Fig. 4). The overall expressional patterns of candidate virulence factors are summarized below.

#### Secondary metabolite (SM) synthetases

The predicted 88 SM synthetases included eight dimethylallyl tryptophan synthases (DMAT), 13 nonribosomal peptide synthases (NRPS), 13 NRPS-like proteins, 38 polyketide synthases (PKS), 11 terpene synthases (TS), one PKS-like protein and four PKS-NRPS hybrids. The total number and number in each category resembled those reported for the *C. fructicola* Nara_gc5 strain [13]. RNA-seq detected the expression of 44 genes (CPM ≥ 10 in all three replicate libraries for IL; or CPM ≥ 1 in all three replicate libraries for CON, or APP, or CIH) (Additional file 1: Figure S7a). Interestingly, 20 genes showed the highest expression level in IL, indicating that SM gene expression was preferentially induced during plant infection. *PKS1*, which catalyzes melanin biosynthesis [36], was strongly up-regulated in APP. *NPS6*, a NRPS contributing to siderophore biosynthesis [37], showed higher-level expression in APP and IL.

Among 52 SM clusters, one cluster showed coordinated and specific expressional up-regulation in IL (Additional file 1: Figure S7b). This was a PKS cluster conserved among several *Colletotrichum* genomes including *C. fructicola* Nara_gc5, *C. gloeosporioides*, *C. fioriniae* and *C. higginsianum*. The homologous cluster in *C. higginsianum* (PKS cluster-6), however, was barely expressed during *Arabidopsis* infection [8].

#### Candidate effector proteins (CEPs)

**Fig. 5:**
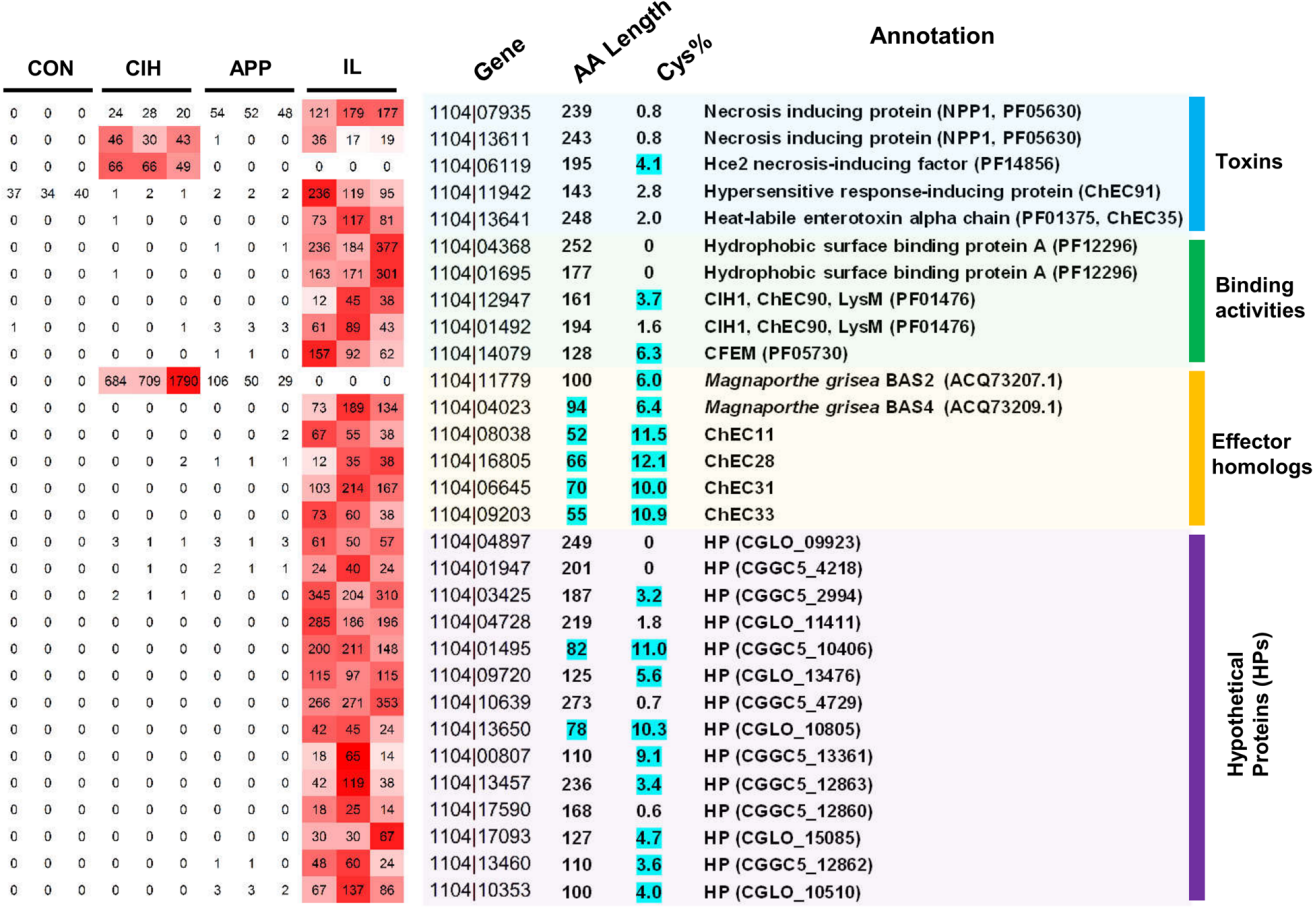
Candidate effector proteins (CEPs) of *C. fructicola*. Short amino acid (AA) length (< 100) and high cysteine content (Cys% > 3%) are highlighted in blue. For each gene, CPM values are indicated

Among 552 predicted small secreted proteins (SSPs), 187 showed significantly different expression among samples. A total of 30 SSP members showed *in planta*-specific expression (CPM being zero or close to zero in CON, APP, and CIH, but at least 10 in IL), or were similar to known fungal effectors, which were named candidate effector proteins (CEPs). Those CEPs were further divided into four categories detailed below (Fig. 5).

The first CEP category contained putative toxins, which included necrosis-inducing proteins (NPP1, PF05630) and Hce2 necrosis-inducing factors (PF14856). The predicted 1104–7 secretome contained nine NPP1s and four Hce2s. RNA-seq detected the expressions of four NPP1s and three Hce2s (CPM ≥ 10 in all three replicate libraries for IL; or CPM ≥ 1 in all three replicate libraries for CON, or APP, or CIH), among which two NPP1s (1104|07935, 1104|13,611) and two Hce2s (1104|06119, 1104|11,817) were differentially expressed. None of the four genes, however, showed *in planta*-specific expression. Interestingly, 1104|13,641, which was homologous to ChEC35 in *Colletotrichum higginsianum* and the bacterial enterotoxin alpha chain, showed strong *in-planta*-specific expression. The ChEC91 homolog 1104|11,942, similar to an *Ophiostoma ulmi* necrosis-inducing protein [38], was highly expressed in CON and IL. Expression of three weak homologues of the host-selective toxin *PtrToxB* [39] was not detected.

The second CEP category contained surface-binding proteins, which included two proteins containing hydrophobic surface binding protein A domain (HsbA, 1104|04368, 1104|01695), two LysM domain-containing proteins (1104|12,947, 1104|01492) and one CFEM domain-containing protein (1104|14,079). All five genes showed *in planta*-specific expression (Fig. 5). The two LysM proteins belong to the CIH1 family, a family of *Colletotrichum* proteins expressed specifically in intracellular biotrophic hyphae and important for fungal virulence [20, 40].

Proteins in the third CEP category lacked obvious domain but showed strong sequence similarity to known biotrophy-associated effectors identified in *C. higginsianum* (ChEC11, ChEC28, ChEC31, ChEC33) or *Magnaporthe grisea* (BAS2, BAS4) (Fig. 5). Except for *BAS2* (1104|11,779), all members showed *in planta*-specific expression. *BAS2* was not detected in IL but was highly expressed in APP and CIH.

Fourteen *in planta*-specific SSPs lacked any function-indicative signature (Fig. 5), among which five (1104|04728, 1104|01495, 1104|10,639, 1104|13,650, 1104|17,590) were specific to the genus *Colletotrichum* based on BlastP searches against the NCBI nr database.

#### CAZYs

Among 1129 predicted CAZY genes, 422 were differentially expressed. These CAZYs were roughly classified into five clusters (Additional file 1: Figure S8a). Cluster1 encompassed genes being strongly induced in APP, which included 27 genes, among which 14 coded for redox enzymes. AA2 (heme-containing peroxidases) and AA5 (copper radical oxidases) showed the strongest enrichments, bonferroni-adjusted *P*-values were 1.4E-08 and 3.7E-05 respectively (hypergeometric tests). Cluster2 genes were highly upregulated in IL; the most strongly enriched family was CE5 (cutinases), with an adjusted *P*-value of 0.02. Cluster3 genes were highly upregulated in CIH; families showing the strongest enrichments functioned in degrading carbohydrate macromolecules. The adjusted enrichment *P*-values for CBM1, AA9 (formerly GH61), PL1, and GH7 were 7.1E-10, 2.3E-05, 0.01 and 0.02 respectively. Cluster5 genes were highly expressed in conidia, the GT frequency for which was the highest among all clusters, and the family showing the strongest enrichment was GH128 (adjusted *P* = 0.001).

Based on CAZY family assignments, 230 CAZY genes were putative PCWDEs and 126 of them were differently expressed (Additional file 1: Figure S8b). Interestingly, 107 of these genes showed the highest expressions in CIH, evidencing strong activation of PCWDEs in CIH.

#### Secreted proteases

Among 96 predicted secretory proteases, 54 members were differentially expressed (Additional file 1: Figure S8c) and 35 showed the highest expression in CIH. Examination of these protease families identified M14As and S53s showing interesting expression patterns. Five of the six predicted M14As showed specific up-regulation in CIH whereas three of the four predicted S53s showed higher expressions in APP and IL (Additional file 1: Figure S8d). The M14A family contains digestive zinc carboxypeptidase activity [41]; S53 family members are characterized by endopeptidase activity with acidic pH optima [42].

### Genes showing infection structure-specific expression or upregulation

#### Conidia

##### Metabolic shunt toward triacylglycerol biosynthesis

**Fig. 6:**
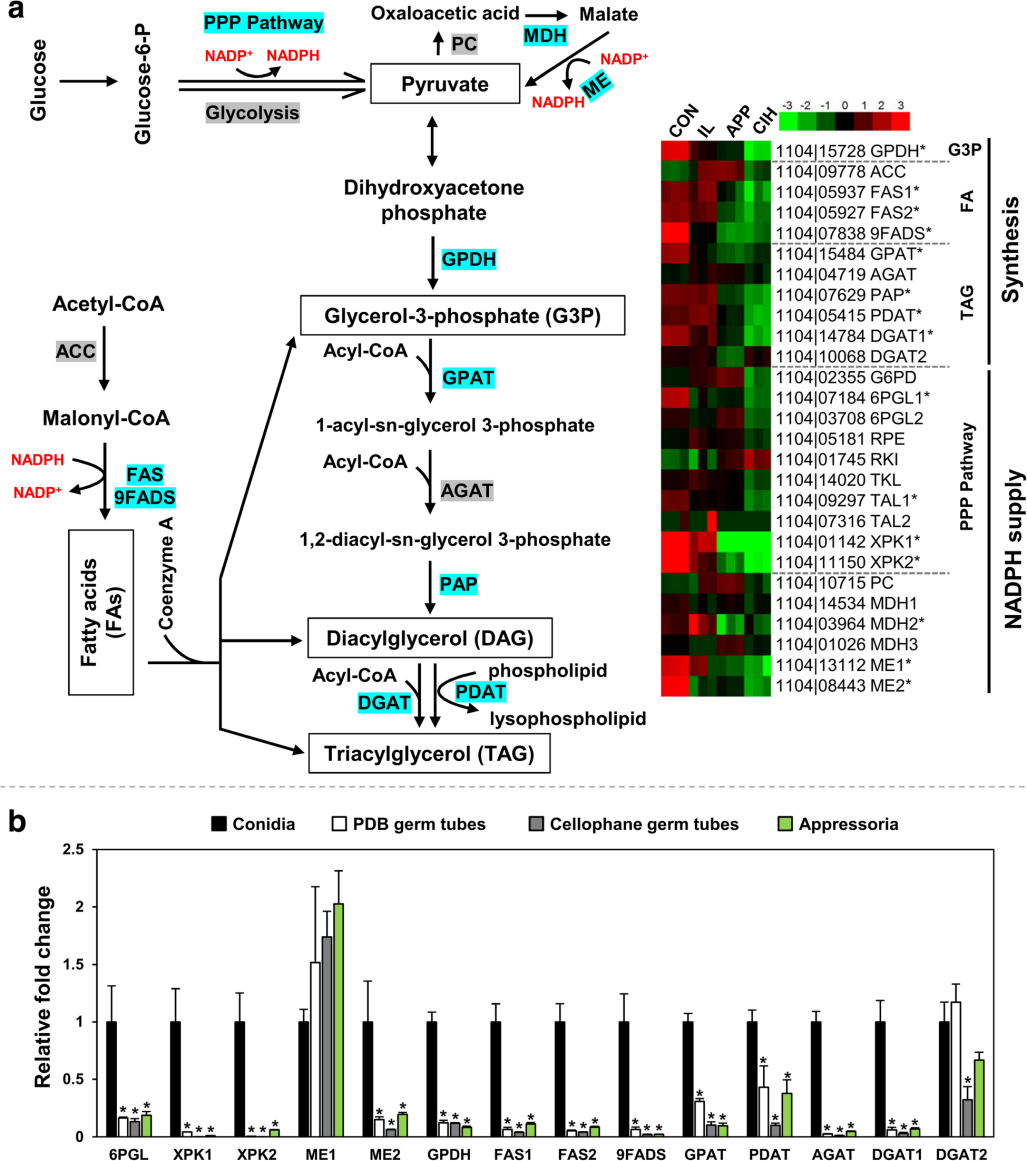
Triacylglycerol (TAG) biosynthetic pathway genes are coordinately up-regulated in conidia. **a** Metabolic pathways leading toward TAG biosynthesis (left) and expressional heatmap of involved genes (right); log2 ratios of expression are plotted. Up-regulated genes (over 2-fold, relative to APP) are highlighted in blue (left) or ‘*’ (right). **b** Expressional validation of selected genes by qRT-PCR. Fold changes relative to conidia were plotted and error bars were calculated from three independent replicates. ‘*’ indicates significant down-regulation relative to CON at *P* < 0.05 using permutation test. ACC: acetyl-CoA carboxylase; AGAT: acylglycerol-phosphate acyltransferase; DGAT: diglyceride acyltransferase; FAS: fatty acid synthase; GPAT: glycerol-3-phosphate O-acyltransferase; GPDH: glycerol-3-phosphate dehydrogenase; MDH: malate dehydrogenase; ME: malic enzyme; PAP: phosphatidic acid phosphohydrolase; PC: pyruvate carboxylase; PDAT: phospholipid:diacylglycerol acyltransferase; PPP: pentose phosphate pathway; 9FADS: Delta-9 fatty acid desaturase

Lipid bodies are important energy reserves in *Colletotrichum* conidia, which are mobilized for energy generation during conidial germination and appressorium formation [43]. A number of genes functioning in lipid biosynthesis were up-regulated in CON. Further examination and qRT-PCR data supported a coordinated up-regulation of genes leading toward triacylglycerol (TAG) biosynthesis (Fig. 6).

Triacylglycerol biosynthesis relies on two metabolic precursors, fatty acids (FAs) and glycerol-3-phosphate (G3P). FA biosynthesis is derived from acetyl-CoA, in the process of which acetyl-CoA carboxylase (ACC) transforms acetyl-CoA into malonyl-CoA, then fatty acid synthetase (FAS) iteratively incorporates malonyl-CoA into a preexisting CoA precursor. The 1104–7 genome contained a single-copy ACC gene (1104|09778), which showed no expressional difference among RNA-seq samples. On the other hand, both copies of FASs (1104|05937, 1104|05927) showed expressional up-regulation in CON relative to APP and CIH. A delta-9 fatty acid desaturase (9FADS, 1104|07838), catalyzing FA desaturation, was also strongly up-regulated in CON, with over 20 folds compared with both APP and CIH. G3P is produced mainly from the C3 metabolite dihydroxyacetone phosphate, being catalyzed by glycerol-3-phosphate dehydrogenase (GPDH). The 1104–7 genome contained a single-copy GPDH (1104|15,728), which was also clearly up-regulated in CON (~ 8 fold relative to APP). The expressional upregulation of FASs, 9FADS, and GPDH in conidia was also validated by qRT-PCR (Fig. 6b).

The enzymatic condensation of FAs and G3P into TAGs is a multi-step process catalyzed by at least five enzymes. The first and rate-limiting step is the formation of 1-acyl-sn-glycerol 3-phosphate from G3P and Acyl-CoA, catalyzed by glycerol-3-phosphate acyltransferase (GPAT) [44]. The single-copy GPAT gene (1104|15,484) showed the strongest expression in CON, approximately 7-fold stronger than APP, and this expressional upregulation was validated by qRT-PCR. The formed 1-acyl-sn-glycerol 3-phosphate would be further acylated into TAG via the coordinated action of acylglycerol-phosphate acyltransferase (AGAT), phosphatidate phosphatase (PAP), phospholipid:diacylglycerol acyltransferase (PDAT), and diglyceride acyltransferases (DGATs). The 1104–7 genome contained five genes encoding these enzymes (AGAT, PAP, PDAT, DGAT1, DGAT2). The expression of PAP, PDAT and DGAT1 was apparently higher in CON than in APP and CIH in RNA-Seq (Fig. 6a). The expression of AGAT, PDAT, DGAT1 and DGAT2 was further assessed by qRT-PCR, in which AGAT, PDAT and DGAT1 were significantly up-regulated in CON (Fig. 6b).

FA biosynthesis and FA desaturation require the reducing force generated from NADPH. In oleaginous fungi, the generation of NADPH has been proposed to be a rate-limiting step during FA biosynthesis, for which pentose phosphate pathway (PPP) and NADP^+^-dependent malic enzyme (ME) play critical roles [45, 46]. Compared with the other three RNA-Seq samples, one 6-phosphogluconolactonase (6PGL1, 1104|07184), one transaldolase (TAL1, 1104|09297), and two xylulose 5-phosphate phosphoketolases (XPK1 [1104|01142], XPK2 [1104|11,150]) were significantly up-regulated in CON. All of these enzymes participate in the PPP pathway. The expressional upregulation of 6PGL1, XPK1, and XPK2 was further validated by qRT-PCR (Fig. 6b). The 1104–7 genome contained two predicted MEs (ME1 [1104|13,112] and ME2 [1104|08443]), both specifically up-regulated in CON based on RNA-Seq (Fig. 6a). However, qRT-PCR validated only the expressional up-regulation of ME2 (Fig. 6b).

Overall, a range of enzymes functioning in TAG enzymatic condensation and the production of TAG condensation precursors (FA, G3P, NADPH) were upregulated in a coordinated manner in CON, supporting a metabolic shunt toward storage lipid biosynthesis in developing conidia.

##### Hypoxia and oxidative stress responses

In filamentous fungi, hypoxia responses are conservatively regulated by transcription factors (TFs) of the sterol regulatory element binding protein (SREBP) class [47], with SrbA and SrbB from *Aspergillus fumigatus* being best characterized [48]. The 1104–7 genome contained 12 SREBPs, including one SrbA homolog and three SrbB homologues (Additional file 1: Figure S9a). All four genes were specifically up-regulated in CON relative to APP, CIH and IL (Additional file 1: Figure S9b). In addition to SREBPs, a range of downstream genes known to be activated by SrbB, or activated by SrbB together with SrbA were also up-regulated in CON (Additional file 1: Figure S9b). Interestingly, no obvious expressional upregulation was observed with genes activated by SrbA alone (Additional file 1: Figure S9b).

The fungal bZIP transcription factor *AP1* is an important oxidative stress response regulator [49–51]. Based on RNA-Seq, the 1104–7 *AP1* gene (1104|15,415) showed four and nine-fold expressional up-regulation in CON compared with APP and CIH, indicating elevated oxidative stress responses in conidia. In accordance, the single-copy alternative oxidase gene (1104|02648), which is important for attenuating mitochondrial ROS production, was highly up-regulated in CON (Additional file 1: Figure S9c), the up-regulation folds were over 100 compared with APP and CIH. Moreover, one mitochondrial MnSOD (1104|08933) and one cytosolic MnSOD (1104|15,641) were strongly up-regulated in CON compared with APP and CIH (Additional file 1: Figure S9c). Additional ROS-detoxifying enzymes, including catalase, glutathione-disulfide reductase, thioredoxin reductase, and glutathione s-transferase, were also highly induced in CON (Additional file 1: Figure S9c). These results supported elevated oxidative stress responses in developing conidia.

##### Environmental signal perceptions

The protein family CFEM (common in fungal extracellular membranes) was enriched among genes up-regulated in CON. CFEM is an eight cysteine-containing domain playing diverse roles including virulence, maintaining cell wall integrity, biofilm formation, and iron acquisition; CFEM-containing proteins have been proposed to function as cell-surface receptors or signal transducers, or as adhesion molecules [52–54]. The 1104–7 genome contained 40 CFEM genes, and the expression of 31 members was significantly different among samples (Additional file 1: Figure S10). Interestingly, up to 16 CFEMs showed the highest expression in CON. A large portion of these CFEMs contained predicted secretory peptides and transmembrane regions, indicating a potential function in environmental signal perception.

#### Appressoria

##### Melanin biosynthesis

**Fig. 7:**
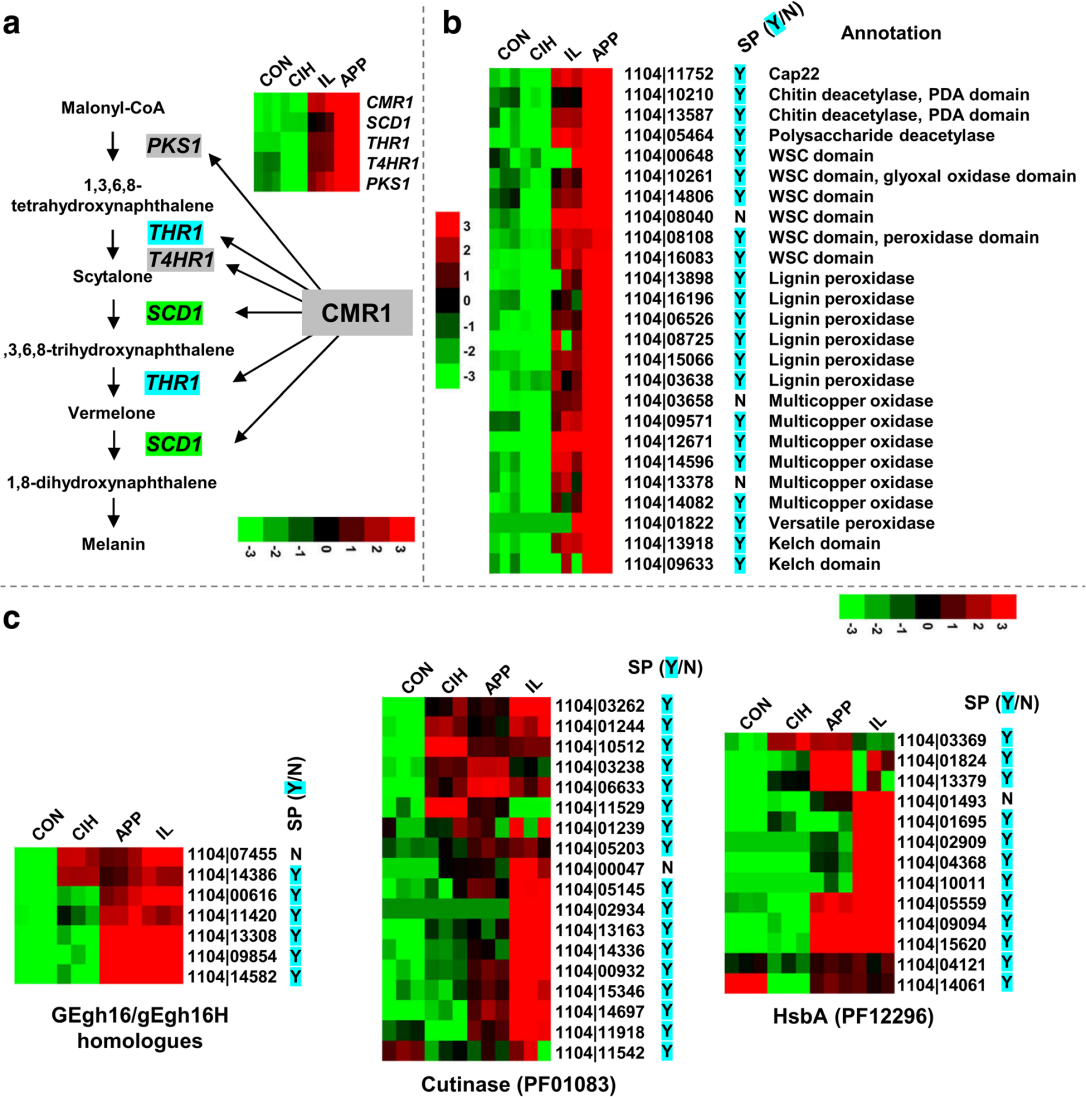
Expression of genes putatively involved in appressorial development and function. **a** 1,8-DHN melanin biosynthetic pathway. **b** Cap22, carbohydrate binding and oxidoreductive enzymes. **c** Genes in the GEgh16/gEgh16H family (left), cutinase family (middle), and HsbA family (right)

In *Colletotrichum*, appressorial melanization is critical for penetration success, and the melanin biosynthetic pathway has been well-characterized. During melanin biosynthesis, malonyl-CoA is first converted into 1,8-dihydroxynaphthalene (1,8-DHN) by four pathway enzymes, namely PKS1, T4HR1, THR1, and SCD1 [55]. The intermediate 1,8-DHN is further polymerized into melanin by laccase [56]. Within expectation, the *C. fructicola PKS1*, *T4HR1*, *THR1*, and *SCD1* genes were all strongly up-regulated in APP (Fig. 7a); the fold increases ranged between 50 to over 250 compared with CON. The *THR1* (1104|11,188) transcript was in fact the most abundant among all genes expressed in APP. In *C. lagenarium*, a Cys2His2 zinc finger transcription factor CMR1 regulates melanin accumulation in mycelia but not in appressoria [57]. Interestingly, the *C. fructicola CMR1* homolog (1104|12,627) showed around 140-fold expressional upregulation in APP compared with CON.

##### Extracellular binding and cell wall modification activities

The *C. gloeosporioides CAP22* gene encodes a 22 kDa protein that is highly induced during appressorium formation [58], and localizes to the appressorial cell wall when it is secreted. The 1104–7 genome contained one single-copy *CAP22* homolog, which was highly up-regulated in APP and its transcript abundance ranked third among all genes. Significantly up-regulated genes in APP also included three genes encoding the polysaccharide deacetylase domain (PF01522) and six genes encoding the carbohydrate-binding WSC domain (PF01822) (Fig. 7b). All of those carbohydrate-binding proteins contained predicted signal peptides except for 1104|08040. Two of the six WSC proteins contained additional domains; 1104|08108 had a haem-like peroxidase domain (PF00141) in the N-terminal region whereas 1104|10,261 had a glyoxal oxidase domain (PF07250) in the C-terminal region.

##### Extracellular oxidoreductive activities

Several redox enzymes (six putative lignin peroxidases, five putative multicopper oxidases, one putative versatile peroxidase, and one putative GMC oxidoreductase) were strongly up-regulated in APP compared with their expression in CON, CIH and IL (Fig. 7b). Eleven of the 13 proteins contained predicted signal peptides, indicating strong extracellular oxidoreductive activity. It is important to note that copper-containing laccase is important for appressorial melanization in *C. orbiculare* [56].

##### Additional activities

*GEgh16*/*gEgh16H* homologues make up a conserved protein family in fungi which play important virulence roles by regulating plant penetration [59–61]. The 1104–7 genome contained seven g*Egh16*/*gEgh16H* homologues; five showed higher expressions in both APP and IL, supporting their relation to infection (Fig. 7c). In particular, the transcript accumulation of 1104|13,308 ranked the 15th in APP whereas the transcript accumulation of 1104|14,582 ranked the seventh in IL. Another gene family related to appressorial function was HsbA (hydrophobic surface binding protein A domain, PF12296). In *Aspergillus oryzae*, a HsbA protein binds hydrophobic surface and recruits lytic enzymes (e.g. cutinases) to promote the degradation of hydrophobic solid materials [62]. The 1104–7 genome encoded 13 HsbA proteins; of these, five showed IL-specific expressional upregulation, two showed expressional APP-specific upregulation, and three showed higher expression in both APP and IL (Fig. 7c). The 1104–7 genome contained 18 predicted cutinases (PF01083). Interestingly, at least 9 genes showed clear IL-specific up-regulation whereas only two showed APP-specific upregulation (Fig. 7c). It is likely that plant-dependent chemical induction is more important than hydrophobicity in triggering cutinase gene expression.

Two functionally unknown proteins containing predicted secretion signal peptides and the protein-binding kelch domain (1104|09633, 1104|13,918) were also strongly up-regulated in APP, with 1104|13,918 being the 14th most abundantly expressed gene in APP.

#### Cellophane infectious growth

*Colletotrichum* appressoria can penetrate cellophane and differentiate bulbous cellophane infectious hyphae (CIH). In appearance, these structures resemble *in planta* biotrophic infectious hyphae, and have been named pseudobiotrophic hyphae [63]. In the present study, however, CIH and IL shared only 10.6% of genes that were significantly co-upregulated compared with APP. Moreover, 1104|12,947 and 1104|01492, two homologues of the *Colletotrichum* biotroph marker *CIH1*, were specifically expressed in IL, but not in CIH. Thus, the global gene expressional profile of CIH differed from IL. Compared with the other tissue types, the most obvious expressional pattern shift observed with CIH was the cooperative up-regulation of PCWDEs and secretory proteases.

In filamentous fungi, the expression of PCWDE genes is tightly regulated. Two major classes of transcription factor, XlnR/Xyr1 and CLR-2/ClrB/ManR, regulate cellulolytic enzyme expressions in Ascomycota fungi [64–66]. The functional importance of these two classes of genes differs among genera; *Trichoderma reesi* relies mainly on XlnR whereas *Aspergillus* spp. and *Neurospora* spp. rely mainly on CLR-2. In *N. crassa*, cellulose-induced expression of CLR-2 requires an additional transcriptional regulator CLR-1 (clrA). The 1104–7 genome contained one CLR-1 homolog (1104|14,699), two CLR-2 homologues (1104|02259, 1104|08827), and two XlnR homologues (1104|09929, 1104|00491). Compared with CON, APP and IL, weak expressional upregulation was observed for the CLR-2 homolog 1104|08827 and the XlnR homolog 1104|09929 in CIH whereas strong expressional up-regulation was observed for the CLR-2 homolog 1104|02259 (11-fold relative to APP) (Additional file 1: Figure S8b).

#### Plant infection

##### Phosphate starvation responses

**Fig. 8:**
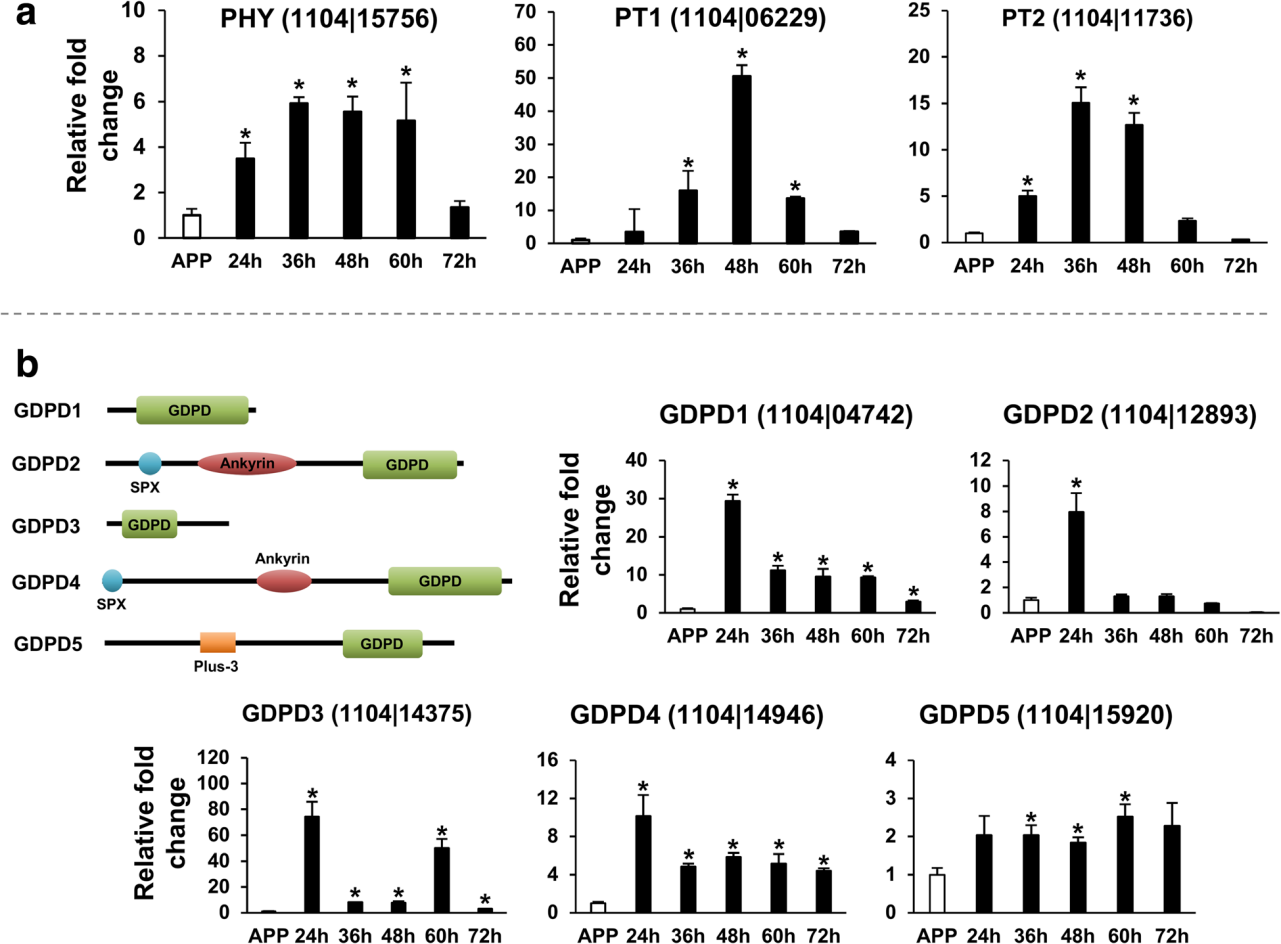
Phosphate starvation responsive genes of *C. fructicola* are activated during the early infection phases. **a** qRT-PCR expressional profiles of one putative phytase and two putative phosphate transporters during apple (cv. Gala) leaf infection. Expression at different times after inoculation (black bars) was normalized to appressoria formed on onion epidermis (APP, white bar); error bars were calculated from three independent replicates. **b** Protein domain structures of the five glycerophosphodiester phosphodiesterase (GDPD) genes (GDPD1 to GDPD5) and their expressional profiles. In (**a**) and (**b**), ‘*’ indicates significant up-regulation relative to APP at *P* < 0.05 using permutation test

Gene Ontology enrichment analyses showed that phosphate transport (GO:0005315) and glycerophosphodiester phosphodiesterase (GO:0008889) activities were significantly enriched among genes up-regulated in IL (Fig. 3), indicating a phosphate-limited *in planta* environment [67]. Using qRT-PCR, we examined the expression of phosphate starvation responsive genes during the infection process of *C. fructicola*. The expression of one phytase homolog (PHY, 1104|15,756) and two putative phosphate transporters (PT1 [1104|06229], PT2 [1104|11,736]) was highly up-regulated in early infection; all peaked between 36 hpi and 48 hpi (Fig. 8a). The 1104–7 genome also encoded five proteins with glycerophosphodiester phosphodiesterase domains (GDPD1–5) (Fig. 8b). GDPD5 expression was relative constant, but the other four GDPDs showed obvious expressional upregulation at 24 hpi. GDPD3 was also up-regulated at 60 hpi. GDPD2 and GDPD4 contained SPX domains (PF03105) at their N-terminal regions. SPX domain proteins are key players regulating inorganic phosphate signaling, particularly phosphate starvation responses [67–70].

##### Utilizing plant-derived quinate as a carbon source

**Fig. 9:**
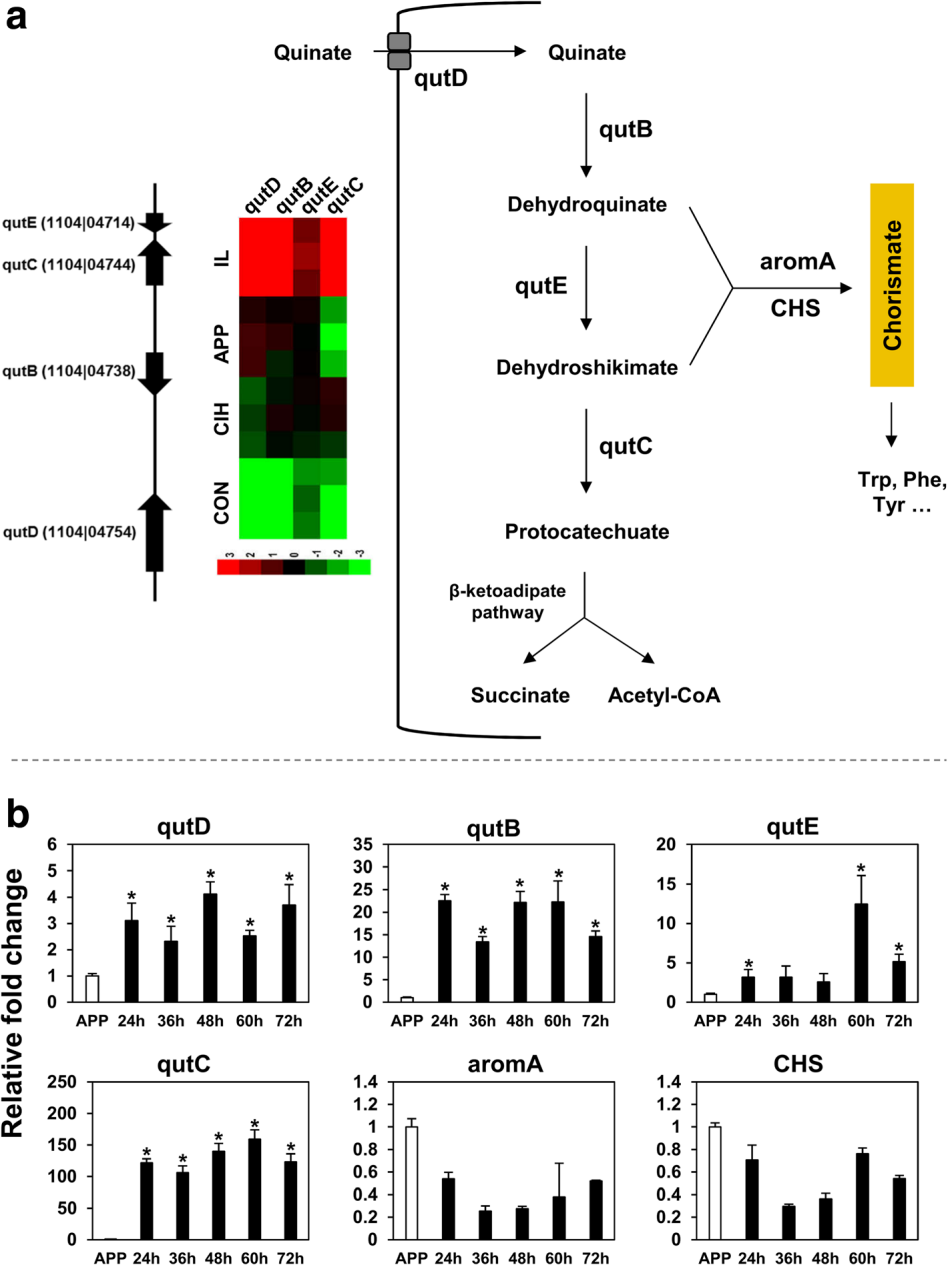
Expressional characteristics of quinate metabolism-related genes during apple (cv. Gala) leaf infection. **a** the quinate gene cluster organization, function, and RNA-seq expression profile. **b** qRT-PCR expressional profiles, ‘*’ indicates significant up-regulation relative to APP at *P* < 0.05 using permutation test

Quinate is a lignin-related aromatic hydrocarbon comprising up to 10% of the total weight of decaying leaf litter [71]. In nature, several fungi can utilize quinate as the sole carbon source, which requires a ‘*qut*’ gene cluster [72]. Genes on the cluster include quinate permease (*qutD*), quinate dehydrogenase (*qutB*), type II 3-dehydroquinase (*qutE*), and 3-dehydroshikimate dehydratase (*qutC*). These enzymes together take up exogenous quinate and transform it into protocatechuate, which can be further catabolized into succinate and acetyl CoA for energy generation via the TCA cycle, or be transformed into chorismate for aromatic compound biosynthesis. The qutC gene catalyzes the rate-limiting step of quinate utilization. Transcription of the ‘*qut*’ genes is typically quinate-inducible. In RNA-seq, the *C. fructicola* homologs of ‘*qut*’ genes showed *in-planta* specific up-regulation (Fig. 9a), which was further validated by qRT-PCR (Fig. 9b). Compared with APP, the expression levels of *qutD*, *qutB*, and *qutC* were significant higher at all infection time points. qutC, in particular, showed over 100-fold expressional upregulation. *qutE* also showed expressional upregulation, but its up-regulation extent was not as obvious as the other three genes (Fig. 9b). These results indicate that during infection, *C. fructicola* actively absorbs and catabolizes extracellular quinate. The transformation of quinate-derived dehydroquinate into chorismite is catalyzed by the penta-functional AROM (1104|03859) and chorismate synthase (1104|06927). Interestingly, neither gene was up-regulated during infection (Fig. 9b), indicating that quinate catabolism is more likely linked with TCA cycle-mediated energy generation than aromatic compound synthesis.

## Discussion

Genome sequencing has revealed a rich repertoire of candidate virulence factors in the *C. fructicola* genome. Lack of data on their expression patterns, however, has limited attempts to prioritize genes for functional validation. In this study, we first compared the global transcriptome profiles of four types of infection-related structures, then examined the expressional changes of SM biosynthetic enzymes, SSPs, CAZYs, and secretory proteases among these structures. We identified a range of *in planta*-specific candidate effector proteins (CEPs), some of which were genus-specific in the NCBI database whereas others were homologues of known CEPs. Our data also revealed 20 SM synthetases showing *in planta* upregulation and one SM gene cluster showing *in planta*-specific expression. These genes are important candidates for identifying regulators of pathogen host interactions. Conidia, appressoria and infectious hyphae are important infection-related structures; a strong transcriptomic shift was observed among these structures, and the differentially expressed genes provide important clues on the pathogen’s morphological transitions during infection.

### Data quality control

Due to the small ratio of pathogen biomass relative to plant host, analyzing pathogen transcriptome during early plant infection can be very challenging. However, these ‘rare’ pathogen-derived RNA-seq reads can provide important insights into the early plant-pathogen interaction dynamics. Recently, Zhang et al. reported the *C. fructicola* transcriptome at three time points post strawberry leaf inoculation [73]. The mapped fungal reads are extremely low for all three stages (0.02 to 0.11 million). However, these reads successfully recover a number of candidate virulence genes such as candidate effectors, and genes functioning in adhesion and appressorium penetration, fatty acid metabolism, and transportation.

In the current study, only small fractions (0.53 to 1.29%) of total IL reads could be mapped to the fungal genome. Even though we generated over 6-fold more reads for the IL libraries (~ 10 Gb each), the mapped reads for the IL libraries (0.16 to 0.42 million) were still less than 10% of other biological conditions (3.15 to 5.45 million). Since library size reduction interferes with the accurate expressional estimation of lowly-expressed genes, we estimated the CPM threshold above which accurate expressional estimation might be achieved, for which we relied on the inter-replicate variation of CPM values. Our data showed that filtering away genes with CPM < 5 could keep the relative CPM error to less than 0.6 for over 95% of genes in CON, APP, and CIH; for the IL samples, CPM threshold of 50 would control the relative error of 96% genes to less than 1.0. These thresholds were incorporated in our further differential expression analyses.

We combined several measures in our analysis procedure to ensure rigorous DEG identification. First, we performed DEG identification with a high fold change threshold. In each pairwise comparison, only genes showing more than 8-fold CPM differences were kept for further statistical testing. Second, both DESeq2 and edgeR were used for DEG identification, and we required the identified DEGs to be supported by both programs. Third, candidate DEGs co-identified by DESeq2 and edgeR were further filtered with the optimized CPM thresholds. Data examination showed that identified DEGs clearly had different CPM values and were enriched with genes being lowly-expressed at one stage but highly activated at another stage. Furthermore, relative fold changes calculated with RNA-Seq and qRT-PCR showed obvious correlation. These results support the reliability of our DEGs identification procedure.

### Distinct transcriptome profiles between in vivo biotrophic hyphae and in vitro cellophane infectious hyphae

*Colletotrichum* pathogens infect hemibitrophically [8], and differentiate bulbous intracellular biotrophic hyphae upon appressorium-mediated penetration. The fact that in vitro-induced cellophane infectious hyphae (CIH) resemble biotrophic hyphae in appearance suggests that the two infection-related structures may share similar global gene expression patterns. In this study, the transcriptome profiles between CIH and infected leaf (IL), however, differed dramatically, sharing only 10.6% of the genes that were upregulated when compared with appressoria. In *C. higginsianum, C. lindemuthianum*, and *C. graminicola*, the LysM domain-containing CIH proteins specifically express at the early biotrophic infection phase, but not at other developmental or infection stages, supporting the interpretation that *CIHs* are biotroph markers [20, 40, 74]. In this study, the two *C. fructicola CIH1* homologues expressed highly in IL, but not in CIH, suggesting that a considerable fraction of infectious hyphae in the IL sample were in biotrophic phase and that infection of the artificial cellophane membrane does not trigger the expression of biotrophy-related genes. CIH1 homologues localize to the surface of intracellular biotrophic hyphae [20, 40], and their LysM domains bind chitin oligomers and suppress chitin-triggered innate immunity [20]. We hypothesize that a plant-derived signal is important for the expressional activation of biotrophy-related genes including *CIH1*.

While evidence supporting its expressional relatedness to biotrophic hyphae is lacking, the transcriptome of cellophane infectious hyphae features highly activated expression of lytic enzymes including PCWDEs and secretory proteases. In *C. higginsianum* and *C. graminicola*, lytic enzymes are strongly activated during necrotrophic colonization and are presumably important for acquiring nutrients from dying host cells [8]. In the future, it would be informative to compare the transcriptome profiles between cellophane infectious hyphae and necrotrophic *in planta* hyphae.

### Genes related to the development of conidia and appressoria

After landing on plant surfaces, pathogenic fungi may suffer from nutrient deprivation during germination and penetration. Some pathogens counteract this potential stress with an avoidance mechanism, by which the spores sense exogenous nutrients and germinate only when exogenous nutrients are available. Others, such as *Colletotrichum* spp., produce energy reserves during spore maturation and mobilize them when the spore germinates. Most *Colletotrichum* conidia contain lipid droplets, and light microscopy shows that these droplets are rapidly consumed during conidial germination [43]. By comparing gene expressional differences between conidia and other fungal structures, we demonstrated in this study that conidial development involves a coordinated expressional upregulation of genes synthesizing the triacylglycerol (TAG) precursors (FAs, G3P), genes generating the NADPH cofactor, and genes functioning in condensing FAs and G3P. These results point toward an active metabolic shunt toward TAG lipid biosynthesis. The transcriptomic data are thus in agreement with the earlier histological observations [43], and together support the importance of storage lipid as an energy reserve in *Colletotrichum* conidia. In the future, generating deletion mutants for genes in the TAG biosynthetic pathway would be valuable for determining its functional importance toward conidial lipid body formation, conidial development, conidial germination and fungal virulence.

In addition to TAG biogenesis, the transcriptome data generated in this study indicated additional cellular activities related to conidial development, i.e., elevated hypoxia responses, elevated oxidative stress responses, and elevated expression of light-perceiving genes. In *Colletotrichum*, O_2_ availability has been reported to affect conidial production. Slininger et al. reported that under fermentation conditions, the specific O_2_ demand of the sporulating mycelia of *C. truncatum* is an order of magnitude less than that of growing mycelia [75]; moreover, 55% or higher dissolved O_2_ tension supports maximum mycelial growth, but reduces sporulation rate [75]. Similarly, our previous study showed that in liquid shake culture, a small culture volume or a slow shake speed dramatically decreases conidial production efficiency in *Colletotrichum* [21]. It appears likely that efficient *Colletotrichum* conidiation requires a tightly-regulated O_2_ exposure, in which process activation of hypoxia-responsive genes plays a regulatory role.

Concomitant with the elevated expression of hypoxia-responsive genes, expression of the oxidative stress regulator AP1 and several ROS-detoxifying enzymes were also activated in conidia. Intuitively, the results appear contradictory given that intracellular ROS generation depends strictly on the presence of O_2._ However, experimental evidence has demonstrated the presence of hypoxic oxidative stress and suggested that increased hypoxic oxidative stress may function as a second messenger and contribute toward better hypoxia adaptation [76, 77]. The ROS-detoxifying related alternative oxidase (AOX) gene, for instance, has been consistently observed to be induced by both hypoxia and treatments with O^2−^-generating compounds [78]. Deleting the cytochrome c (CycA) gene in *A. fumigatus* caused reduced hypoxic growth and concomitantly higher ROS resistance; it is likely that a disturbance of electron transport chain-related ROS homeostasis affects hypoxic adaptation [79]. In this study, the *C. fructicola* AOX gene was also strongly upregulated in conidia, supporting the speculation that the observed activation of oxidative stress responses is hypoxia-related.

Appressoria are specialized infection structures critical for host epidermis penetration. As expected, several genes known to be associated with appressorial development were highly expressed in the APP sample, including the dihydroxynaphthalene (DHN) melanin biosynthesis pathway, an ortholog of the *Colletotrichum gloeosporioides* CAP22, and several GEgh16/gEgh16H homologues. The appressorial transcriptome was also featured by a highly induced expression of genes with putative carbohydrate binding or oxidoreductive functions, such as chitin deacetylases, WSC proteins, lignin peroxidases, and multicopper oxidases. Most of these proteins contained predicted secretory peptides, indicating their extracellular localization. These proteins may contribute toward appressorial development or penetration via cell wall modification (e.g. chitin deacetylases, WSC proteins, oxidases, Cap22), surface attachment (e.g. HsbA), or substrate hydrolysis (e.g. lignin peroxidase). A recent study by Zhang et al. [73] shows that *C. fructicola* genes encoding HsbAs, cutinases, GEgh16/gEgh16H homologues (GAS1), and DHN melanin biosynthetic enzymes are also significantly up-regulated at the early phase of strawberry infection (24 hpi). The co-upregulation of these genes in independent studies supports their functional importance in regulating plant penetration.

### Features of the *in planta* environment encountered during *C. fructicola* colonization

Filamentous plant pathogens establish infection by secreting effector proteins which manipulate plant cellular activities and suppress plant immune responses. In this study, 30 candidate effector proteins (CEPs) were identified from *C. fructicola*, with the expression of many members being strongly induced during apple leaf infection. Interestingly, several up-regulated CEPs (e.g. 1104|01495, 1104|10,639, 1104|06645) were also significantly up-regulated during strawberry infection [73]. Interestingly, ChEC31, which is the CEP homolog of *C. fructicola* 1104|06645 in *Colletotrichum higginsianum*, also shows expressional upregulation during *Arabidopsis* leaf infection [14]. Thus, ChEC31 represents a CEP being conservatively up-regulated in at least three *Colletotrichum* pathosystems. On the other hand, there are also CEPs (e.g. ChEC6, ChCE30, ChEC44) showing *in-planta* expressional upregulation in both *C. fructicola*-strawberry and *C. higginsianum*-*Arabidopsis* interactions [14, 73], but being lowly expressed in *C. fructicola*-apple leaf interaction. ChEC6, in particular, was not even detected to be expressed in our study although it is one of the most induced genes in the other two pathosystems [14, 73]. *C. fructicola* has a broad host range, but there has been study indicating that this species contains individual host-limited forms [2]. In the future, it would be interesting to compare the expression difference of *C. fructicola* CEPs among isolates derived from different hosts.

In addition to the activation of candidate virulence genes including CEPs, PCWDEs, proteases, SM synthases, transporters and P450s, data presented in this study suggest that plant colonization by *C. fructicola* involves active transcription of phosphate starvation regulators (glycerophosphodiester phosphodiesterase domains, GDPDs) and phosphate absorption-related genes (phytase, phosphate transporters). Four of the five GDPDs showed obvious expressional up-regulation at the very early colonization phase (24 hpi). Importantly, two of the up-regulated GDPDs also contained the SPX domain, a phosphate starvation-related domain playing key roles in regulating phosphate homeostasis [70, 80]. In *E. coli*, expression of GDPDs is phosphate starvation-responsive [67]. In this study, one phytase and two phosphate transporters (PT1, PT2) also showed expressional upregulation during apple leaf infection, but were highest at late biotrophic phase (36–48 hpi). It seems that the biotrophic colonization of *C. fructicola* features an activated phosphate starvation response, which involves a rapid expressional activation of GDPDs and an ensuing expressional activation of phosphate absorption-related genes. The existence of this phosphate starvation response would further suggest a phosphate-limited *in planta* environment associated with the biotrophic colonization of *C. fructicola*. Interestingly, activation of phosphate starvation response has been demonstrated in two additional cases of *Colletotrichum*-plant interactions. In *C. graminicola*, phytase (acidic phosphatase) and phosphate transporters are actively expressed in biotrophic infectious hyphae [74]. In the *Arabidopsis* endophyte *C*. *tofieldiae*, fungal colonization promotes phosphorus transfer from soil to plant, and enhances phosphate starvation resistance [81]. In the future, gene functional studies are needed to determine whether this phosphate starvation response is a conserved mechanism regulating plant interactions of *C. fructicola* and other *Colletotrichum* species.

Many fungi can utilize plant-derived quinate as a carbon source, which requires a cluster of quinate utilization genes. In *Aspergillus* spp. and *Neurospora* spp., the expression of quinate utilization genes is subject to quinate substrate induction [72, 82]. In this study, the expression of *C. fructicola* genes in the quinate utilization cluster showed clear *in planta* up-regulation, indicating elevated *in planta* quinate availability. Quinate is a metabolite intermediate interconnected with chorismite, another metabolite important for the biosynthesis of aromatic defense compounds such as salicyclic acid (SA), flavonoids, and phenylpropanoids. The biosynthesis of both chorismite and quinate utilize dehydroquinate as a precursor, and it has been proposed that hemibiotrophic pathogens divert host dehydroquinate metabolite toward quinate production, and away from aromatic defense compound production during infection [83]. In this study, three of the four *C. fructicola* quinate cluster genes (*qutD*, *qutB*, *qutC*) showed significant expressional upregulation during the entire process of apple leaf infection. In another *Colletotrichum* fungus *C. orbiculare*, however, the expression of quinate cluster genes peak at late necrotrophic phase during tobacco infection [13]. These results indicate that *Colletotrichum* infection elicits an elevated *in planta* quinate availability, whereas the relevance of such increase to plant defense suppression requires further examination.

## Conclusions

This study represents a systemic transcriptomic analysis of several infection-related structures of *C. fructicola*. The identified structure-specific genes and candidate virulence genes provide clues regarding mechanisms regulating infection-related developments and plant host interactions. Data reported in this study provide important reference for further efforts aimed at revealing fungal infection mechanisms and identifying disease control targets.

## Additional files

Additional file 1 **Table S1**. Bioinformatic command lines used in this study. **Table S2**. qRT-PCR primers used in this study. **Table S3**. Summary statistics of the reads mapping outcomes for individual RNA-Seq libraries. **Figure S1**. Steps of identifying differentially expressed genes. **Figure S2**. Saturation analysis of HTSeq reads count dataset. **Figure S3**. Defining the criterion for filtering lowly expressed genes. **Figure S4**. Density plot showing the effect of gene filtering on the distribution of relative CPM errors for the CIH (top) and IL (bottom) conditions. **Figure S5**. Identification of differentially expressed genes. **Figure S6**. CPM distribution patterns of identified DEGs. **Figure S7**. Differentially expressed secondary metabolite (SM) synthetase genes and a SM gene cluster showing in planta-specific expression. **Figure S8**. Differentially expressed CAZY genes and secreted proteases. **Figure S9.** Hypoxia and oxidative stress-responsive genes are up-regulated in conidia. **Figure S10.** Protein domain organization and gene expression patterns of putative CFEM proteins. (PDF 1307 kb)

Additional file 2 **Table S4**. Differently expressed genes identified in this study. (XLSX 813 kb)

## Abbreviations

ACC: Acetyl-CoA carboxylase
APP: Appressoria
CEP: Candidate effector proteins
CFEM: Common in fungal extracellular membranes
CGSC: *Colletotrichum gloeosporioides* species complex
CIH: Cellophane infectious hyphae
CON: Conidia
CPM: Counts per million
DEG: Differently expressed gene
DMAT: Dimethylallyl tryptophan synthases
FA: Fatty acid
FAS: Fatty acid synthetase
G3P: Glycerol-3-phosphate
GDPD: Glycerophosphodiester phosphodiesterase domains
GLS: Glomerella leaf spot
IL: Infected Leaf
LFC: Log fold change
ME: Malic enzyme
NRPS: Nonribosomal peptide synthetase
PCWDE: Plant cell wall degrading enzyme
PKS: Polyketide synthase
PPP: Pentose phosphate pathway
SM: Secondary metabolite
SREBP: Sterol regulatory element binding protein
TAG: Triacylglycerol
TS: Terpene synthases

## References

[1] Silva DN, Talhinhas P, Cai L, Manuel L, Gichuru EK, Loureiro A (2012). Host-jump drives rapid and recent ecological speciation of the emergent fungal pathogen *Colletotrichum kahawae*. Mol Ecol.

[2] Rockenbach MF, Velho AC, Gonçalves AE, Mondino PE, Alaniz SM, Stadnik MJ (2016). Genetic structure of *Colletotrichum fructicola* associated to apple bitter rot and Glomerella leaf spot in southern Brazil and Uruguay. Phytopathology.

[3] Weir BS, Johnston PR, Damm U (2012). The *Colletotrichum gloeosporioides* species complex. Stud Mycol.

[4] Rojas EI, Rehner SA, Samuels GJ, Van Bael SA, Herre EA, Cannon P, Chen R, Pang J, Wang R, Zhang Y et al: *Colletotrichum gloeosporioides* s.L. associated with *Theobroma cacao* and other plants in Panamá: multilocus phylogenies distinguish host-associated pathogens from asymptomatic endophytes. Mycologia 2010, 102(6):1318–1338. 10.3852/09-244 20943565

[5] Prihastuti H, Cai L, Chen H, McKenzie E, Hyde K (2009). Characterization of *Colletotrichum* species associated with coffee berries in northern Thailand. Fungal Divers.

[6] Fungal databases, systematic mycology and microbiology laboratory, ARS, USDA. https://nt.ars-grin.gov/fungaldatabases/index.cfm. Accessed 3 October 2017.

[7] Li H, Zhou G-Y, Liu J-A, Xu J (2016). Population genetic analyses of the fungal pathogen *Colletotrichum fructicola* on tea-oil trees in China. PLoS One.

[8] O'Connell RJ, Thon MR, Hacquard S, Amyotte SG, Kleemann J, Torres MF, Damm U, Buiate EA, Epstein L, Alkan N et al: Lifestyle transitions in plant pathogenic *Colletotrichum* fungi deciphered by genome and transcriptome analyses. Nat Genet 2012, 44(9):1060–1065. 10.1038/ng.2372 PMC9754331 22885923

[9] Münch S, Lingner U, Floss DS, Ludwig N, Sauer N, Deising HB (2008). The hemibiotrophic lifestyle of *Colletotrichum* species. J Plant Physiol.

[10] Crouch J, O’Connell R, Gan P, Buiate E, Torres MF, Beirn L, Shirasu K, Vaillancourt L, Dean AR, Lichens-Park A, Kole C (2014). The genomics of *Colletotrichum*. Genomics of plant-associated Fungi: monocot pathogens.

[11] Baroncelli R, Amby DB, Zapparata A, Sarrocco S, Vannacci G, Le Floch G, Harrison RJ, Holub E, Sukno SA, Sreenivasaprasad S et al: Gene family expansions and contractions are associated with host range in plant pathogens of the genus *Colletotrichum*. BMC Genomics 2016, 17(1):555. 10.1186/s12864-016-2917-6 PMC4974774 27496087

[12] Gan P, Narusaka M, Kumakura N, Tsushima A, Takano Y, Narusaka Y (2016). Genus-wide comparative genome analyses of *Colletotrichum* species reveal specific gene family losses and gains during adaptation to specific infection lifestyles. Genome Biol Evol.

[13] Gan P, Ikeda K, Irieda H, Narusaka M, O’Connell RJ, Narusaka Y (2013). Comparative genomic and transcriptomic analyses reveal the hemibiotrophic stage shift of *Colletotrichum* fungi. New Phytol.

[14] Kleemann J, Rincon-Rivera LJ, Takahara H, Neumann U, Themaat EVL, Does HC (2012). Sequential delivery of host-induced virulence effectors by appressoria and intracellular hyphae of the phytopathogen *Colletotrichum higginsianum*. PLoS Pathog.

[15] Alkan N, Friedlander G, Ment D, Prusky D, Fluhr R (2015). Simultaneous transcriptome analysis of *Colletotrichum gloeosporioides* and tomato fruit pathosystem reveals novel fungal pathogenicity and fruit defense strategies. New Phytol.

[16] Horbach R, Graf A, Weihmann F, Antelo L, Mathea S, Liermann JC, Opatz T, Thines E, Aguirre J, Deising HB (2009). Sfp-type 4′-phosphopantetheinyl transferase is indispensable for fungal pathogenicity. Plant Cell.

[17] Yoshino K, Irieda H, Sugimoto F, Yoshioka H, Okuno T, Takano Y (2012). Cell death of *Nicotiana benthamiana* is induced by secreted protein NIS1 of *Colletotrichum orbiculare* and is suppressed by a homologue of CgDN3. Mol Plant-Microbe Interact.

[18] Bhadauria V, Banniza S, Vandenberg A, Selvaraj G, Wei Y (2013). Overexpression of a novel biotrophy-specific *Colletotrichum truncatum* effector, CtNUDIX, in hemibiotrophic fungal phytopathogens causes incompatibility with their host plants. Eukaryot Cell.

[19] Vargas WA, Sanz-Martín JM, Rech GE, Armijos-Jaramillo VD, Rivera LP, Echeverria MM (2015). A fungal effector with host nuclear localization and DNA-binding properties is required for maize anthracnose development. Mol Plant-Microbe Interact.

[20] Takahara H, Hacquard S, Kombrink A, Hughes HB, Halder V, Robin GP, Hiruma K, Neumann U, Shinya T, Kombrink E et al: *Colletotrichum higginsianum* extracellular LysM proteins play dual roles in appressorial function and suppression of chitin-triggered plant immunity. New Phytol 2016, 211(4):1323–1337. 10.1111/nph.13994 27174033

[21] Wang W, Liang X, Zhang R, Gleason ML, Sun G (2017). Liquid shake culture overcomes solid plate culture in inducing conidial production of *Colletotrichum* isolates. Australas Plant Pathol.

[22] MacManes MD (2014). On the optimal trimming of high-throughput mRNA sequence data. Front Genet.

[23] Langmead B, Trapnell C, Pop M, Salzberg SL (2009). Ultrafast and memory-efficient alignment of short DNA sequences to the human genome. Genome Boil.

[24] Kim D, Pertea G, Trapnell C, Pimentel H, Kelley R, Salzberg SL (2013). TopHat2: accurate alignment of transcriptomes in the presence of insertions, deletions and gene fusions. Genome Biol.

[25] Anders S, Pyl PT, Huber W (2015). HTSeq—a Python framework to work with high-throughput sequencing data. Bioinformatics.

[26] Tarazona S, Furió-Tarí P, Turrà D, Pietro AD, Nueda MJ, Ferrer A, Conesa A (2015). Data quality aware analysis of differential expression in RNA-seq with NOISeq R/bioc package. Nucleic Acids Res.

[27] Anders S, Huber W (2010). Differential expression analysis for sequence count data. Genome Biol.

[28] Robinson MD, McCarthy DJ, Smyth GK (2010). EdgeR: a bioconductor package for differential expression analysis of digital gene expression data. Bioinformatics.

[29] Liang X, Wang B, Dong Q, Li L, Rollins JA, Zhang R, Sun G (2018). Pathogenic adaptations of *Colletotrichum* fungi revealed by genome wide gene family evolutionary analyses. PLoS One.

[30] Jones P, Binns D, Chang HY, Fraser M, Li W, McAnulla C, McWilliam H, Maslen J, Mitchell A, Nuka G et al: InterProScan 5: genome-scale protein function classification. Bioinformatics 2014, 30(9):1236–1240. 10.1093/bioinformatics/btu031 PMC3998142 24451626

[31] de Hoon MJL, Imoto S, Nolan J, Miyano S (2004). Open source clustering software. Bioinformatics.

[32] Saldanha AJ (2004). Java Treeview—extensible visualization of microarray data. Bioinformatics.

[33] Pathan M, Keerthikumar S, Ang C-S, Gangoda L, Quek CYJ, Williamson NA, Mouradov D, Sieber OM, Simpson RJ, Salim A et al: FunRich: an open access standalone functional enrichment and interaction network analysis tool. Proteomics 2015, 15(15):2597–2601. 10.1002/pmic.201400515 25921073

[34] Schmittgen TD, Livak KJ (2008). Analyzing real-time PCR data by the comparative CT method. Nat Protocols.

[35] Hothorn T, Hornik K, van de Wiel MA, Zeileis A. Implementing a class of permutation pests: the coin package. J Stat Softw. 2008;28(8)

[36] Takano Y, Kubo Y, Shimizu K, Mise K, Okuno T, Furusawa I (1995). Structural analysis of PKS1, a polyketide synthase gene involved in melanin biosynthesis in *Colletotrichum lagenarium*. Mol Gen Genet.

[37] Albarouki E, Schafferer L, Ye FH, von Wiren N, Haas H, Deising HB (2014). Biotrophy-specific downregulation of siderophore biosynthesis in *Colletotrichum graminicola* is required for modulation of immune responses of maize. Mol Microbiol.

[38] Yang D, Hubbes M, JRS J, Hubbes M (1994). A glycoprotein isolated from culture filtrates of *Ophiostoma ulmi* as a mansonone-inducing elicitor on elm callus. Mycol Res.

[39] Martinez JP, Oesch NW, Ciuffetti LM (2004). Characterization of the multiple-copy host-selective toxin gene, ToxB, in pathogenic and nonpathogenic isolates of *Pyrenophora tritici-repentis*. Mol Plant-Microbe Interact.

[40] Perfect SE, Pixton KL, O’Connell RJ, Green JR (2000). The distribution and expression of a biotrophy-related gene, CIH1, within the genus *Colletotrichum*. Mol Plant Pathol.

[41] Makarova KS, Grishin NV (1999). The Zn-peptidase superfamily: functional convergence after evolutionary divergence. J Mol Biol.

[42] Reichard U, Léchenne B, Asif AR, Streit F, Grouzmann E, Jousson O, Monod M (2006). Sedolisins, a new class of secreted proteases from *Aspergillus fumigatus* with endoprotease or tripeptidyl-peptidase activity at acidic pHs. Appl Environ Microbiol.

[43] Barbosa AC, AEd C, Graf L, Tomaz R, CFd S, Mendes J, Randi MAF, Buchi D, Schadeck RJG (2006). Morphology and lipid body and vacuole dynamics during secondary conidia formation in *Colletotrichum acutatum*: laser scanning confocal analysis. Can J Microbiol.

[44] Wendel AA, Lewin TM, Coleman RA (2009). Glycerol-3-phosphate acyltransferases: rate limiting enzymes of triacylglycerol biosynthesis. Biochim Biophys Acta Mol Cell Biol Lipids.

[45] Dey P, Mall N, Chattopadhyay A, Chakraborty M, Maiti MK (2014). Enhancement of lipid productivity in oleaginous *Colletotrichum* fungus through genetic transformation using the yeast CtDGAT2b gene under model-optimized growth condition. PLoS One.

[46] Wasylenko TM, Ahn WS, Stephanopoulos G (2015). The oxidative pentose phosphate pathway is the primary source of NADPH for lipid overproduction from glucose in *Yarrowia lipolytica*. Metab Eng.

[47] Bien CM, Espenshade PJ (2010). Sterol regulatory element binding proteins in fungi: hypoxic transcription factors linked to pathogenesis. Eukaryot Cell.

[48] Chung D, Barker BM, Carey CC, Merriman B, Werner ER, Lechner BE, Dhingra S, Cheng C, Xu W, Blosser SJ et al: ChIP-seq and in vivo transcriptome analyses of the *Aspergillus fumigatus* SREBP SrbA reveals a new regulator of the fungal hypoxia response and virulence. PLoS Pathog 2014, 10(11):e1004487. 10.1371/journal.ppat.1004487 PMC4223079 25375670

[49] Sun Y, Wang Y, Tian C (2016). bZIP transcription factor CgAP1 is essential for oxidative stress tolerance and full virulence of the poplar anthracnose fungus *Colletotrichum gloeosporioides*. Fungal Genet Biol.

[50] Lev S, Hadar R, Amedeo P, Baker SE, Yoder OC, Horwitz BA (2005). Activation of an AP1-like transcription factor of the maize pathogen *Cochliobolus heterostrophus* in response to oxidative stress and plant signals. Eukaryot Cell.

[51] Guo M, Chen Y, Du Y, Dong Y, Guo W, Zhai S, Zhang H, Dong S, Zhang Z, Wang Y et al: The bZIP transcription factor MoAP1 mediates the oxidative stress response and is critical for pathogenicity of the rice blast fungus *Magnaporthe oryzae*. PLoS Pathog 2011, 7(2):e1001302. 10.1371/journal.ppat.1001302 PMC3044703 21383978

[52] Kulkarni RD, Kelkar HS, Dean RA (2003). An eight-cysteine-containing CFEM domain unique to a group of fungal membrane proteins. Trends Biochem Sci.

[53] Pérez A, Pedrós B, Murgui A, Casanova M, López-Ribot JL, Martínez JP (2006). Biofilm formation by *Candida albicans* mutants for genes coding fungal proteins exhibiting the eight-cysteine-containing CFEM domain. FEMS Yeast Res.

[54] Ding C, Vidanes GM, Maguire SL, Guida A, Synnott JM, Andes DR, Butler G (2011). Conserved and divergent roles of Bcr1 and CFEM proteins in *Candida parapsilosis* and *Candida albicans*. PLoS One.

[55] Ludwig N, Lohrer M, Hempel M, Mathea S, Schliebner I, Menzel M, Kiesow A, Schaffrath U, Deising HB, Horbach R (2014). Melanin is not required for turgor generation but enhances cell-wall rigidity in appressoria of the corn pathogen *Colletotrichum graminicola*. Mol Plant-Microbe Interact.

[56] Lin SY, Okuda S, Ikeda K, Okuno T, Takano Y (2012). LAC2 encoding a secreted laccase is involved in appressorial melanization and conidial pigmentation in *Colletotrichum orbiculare*. Mol Plant-Microbe Interact.

[57] Tsuji G, Kenmochi Y, Takano Y, Sweigard J, Farrall L, Furusawa I, Horino O, Kubo Y (2000). Novel fungal transcriptional activators, Cmr1p of *Colletotrichum lagenarium* and Pig1p of *Magnaporthe grisea*, contain Cys2His2 zinc finger and Zn(II)2Cys6 binuclear cluster DNA-binding motifs and regulate transcription of melanin biosynthesis genes in a developmentally specific manner. Mol Microbiol.

[58] Hwang CS, Kolattukudy PE (1995). Isolation and characterization of genes expressed uniquely during appressorium formation by *Colletotrichum gloeosporioides* conidia induced by the host surface wax. Mol Gen Genet.

[59] Grell MN, Mouritzen P, Giese H (2003). A *Blumeria graminis* gene family encoding proteins with a C-terminal variable region with homologues in pathogenic fungi. Gene.

[60] Lu J-P, Liu T-B, Lin F-C (2005). Identification of mature appressorium-enriched transcripts in *Magnaporthe grisea*, the rice blast fungus, using suppression subtractive hybridization. FEMS Microbiol Lett.

[61] Xue C, Park G, Choi W, Zheng L, Dean RA, Xu J-R (2002). Two novel fungal virulence genes specifically expressed in appressoria of the rice blast fungus. Plant Cell.

[62] Ohtaki S, Maeda H, Takahashi T, Yamagata Y, Hasegawa F, Gomi K, Nakajima T, Abe K (2006). Novel hydrophobic surface binding protein, HsbA, produced by *Aspergillus oryzae*. Appl Environ Microbiol.

[63] Irieda H, Maeda H, Akiyama K, Hagiwara A, Saitoh H, Uemura A, Terauchi R, Takano Y (2014). *Colletotrichum orbiculare* secretes virulence effectors to a biotrophic interface at the primary hyphal neck via exocytosis coupled with SEC22-mediated traffic. Plant Cell.

[64] Kunitake E, Kobayashi T (2017). Conservation and diversity of the regulators of cellulolytic enzyme genes in Ascomycete fungi. Curr Genet.

[65] Coradetti ST, Craig JP, Xiong Y, Shock T, Tian C, Glass NL (2012). Conserved and essential transcription factors for cellulase gene expression in ascomycete fungi. Proc Natl Acad Sci U S A.

[66] Gielkens MMC, Dekkers E, Visser J, de Graaff LH (1999). Two cellobiohydrolase-encoding genes from *Aspergillus niger* require d-xylose and the xylanolytic transcriptional activator XlnR for their expression. Appl Environ Microbiol.

[67] Ohshima N, Yamashita S, Takahashi N, Kuroishi C, Shiro Y, Takio K (2008). *Escherichia coli* cytosolic glycerophosphodiester phosphodiesterase (UgpQ) requires Mg2+, Co2+, or Mn2+ for its enzyme activity. J Bacteriol.

[68] Duan K, Yi K, Dang L, Huang H, Wu W, Wu P (2008). Characterization of a sub-family of *Arabidopsis* genes with the SPX domain reveals their diverse functions in plant tolerance to phosphorus starvation. The Plant J.

[69] Lee M, O'Regan S, Moreau JL, Johnson AL, Johnston LH, Goding CR (2000). Regulation of the Pcl7–Pho85 cyclin–cdk complex by Pho81. Mol Microbiol.

[70] Secco D, Wang C, Arpat BA, Wang Z, Poirier Y, Tyerman SD, Wu P, Shou H, Whelan J (2012). The emerging importance of the SPX domain-containing proteins in phosphate homeostasis. New Phytol.

[71] Hawkins AR, Giles NH, Kinghorn JR (1982). Genetical and biochemical aspects of quinate breakdown in the filamentous fungus *Aspergillus nidulans*. Biochem Genet.

[72] Lamb HK, Hawkins AR, Smith M, Harvey IJ, Brown J, Turner G, Roberts CF (1990). Spatial and biological characterisation of the complete quinic acid utilisation gene cluster in *Aspergillus nidulans*. Mol Gen Genet.

[73] Zhang L, Huang X, He C, Zhang Q-Y, Zou X, Duan K, Gao Q (2018). Novel fungal pathogenicity and leaf defense strategies are revealed by simultaneous transcriptome analysis of *Colletotrichum fructicola* and strawberry infected by this fungus. Front Plant Sci.

[74] Torres MF, Ghaffari N, Buiate EAS, Moore N, Schwartz S, Johnson CD, Vaillancourt LJ (2016). A *Colletotrichum graminicola* mutant deficient in the establishment of biotrophy reveals early transcriptional events in the maize anthracnose disease interaction. BMC Genomics.

[75] Slininger PJ, Silman RW, Jackson MA (1993). Oxygen delivery requirements of *Colletotrichum truncatum* during germination, vegetative growth, and sporulation. Appl Microbiol Biotechnol.

[76] Dirmeier R, O'Brien KM, Engle M, Dodd A, Spears E, Poyton RO (2002). Exposure of yeast cells to anoxia induces transient oxidative stress: implications for the induction of hypoxic genes. J Biol Chem.

[77] Solaini G, Baracca A, Lenaz G, Sgarbi G (2010). Hypoxia and mitochondrial oxidative metabolism. Biochim Biophys Acta Bioenerg.

[78] Hillmann F, Shekhova E, Kniemeyer O (2015). Insights into the cellular responses to hypoxia in filamentous fungi. Curr Genet.

[79] Grahl N, Dinamarco TM, Willger SD, Goldman GH, Cramer RA (2012). *Aspergillus fumigatus* mitochondrial electron transport chain mediates oxidative stress homeostasis, hypoxia responses and fungal pathogenesis. Mol Microbiol.

[80] Secco D, Wang C, Shou H, Whelan J (2012). Phosphate homeostasis in the yeast *Saccharomyces cerevisiae*, the key role of the SPX domain-containing proteins. FEBS Lett.

[81] Hacquard S, Kracher B, Hiruma K, Münch PC, Garrido-Oter R, Thon MR, Weimann A, Damm U, Dallery J-F, Hainaut M et al: Survival trade-offs in plant roots during colonization by closely related beneficial and pathogenic fungi. Nat Commun 2016, 7:11362. 10.1038/ncomms11362 PMC4859067 27150427

[82] Patel VB, Schweizer M, Dykstra CC, Kushner SR, Giles NH (1981). Genetic organization and transcriptional regulation in the qa gene cluster of *Neurospora crassa*. Proc Natl Acad Sci U S A.

[83] Parker D, Beckmann M, Zubair H, Enot DP, Caracuel-Rios Z, Overy DP, Snowdon S, Talbot NJ, Draper J (2009). Metabolomic analysis reveals a common pattern of metabolic re-programming during invasion of three host plant species by *Magnaporthe grisea*. The Plant J..

