# Supplementary material for: Transcriptomic analysis reveals candidate genes regulating development and host interactions of Colletotrichum fructicola
Source: BMC Genomics. 2018 Jul 28;19:557. doi: 10.1186/s12864-018-4934-0 (PMC6064131; doi:10.1186/s12864-018-4934-0)
Supplement: Supplementary file 1 — Table S1. Bioinformatic command lines used in this study. Table S2. qRT-PCR primers used in this study. Table S3. Summary statistics of the reads mapping outcomes for individual RNA-Seq libraries. Figure S1. Steps of identifying differentially expressed genes. Figure S2. Saturation analysis of HTSeq reads count dataset. Figure S3. Defining the criterion for filtering lowly expressed genes. Figure S4. Density plot showing the effect of gene filtering on the distribution of relative CPM errors for the CIH (top) and IL (bottom) conditions. Figure S5. Identification of differentially expressed genes. Figure S6. CPM distribution patterns of identified DEGs. Figure S7. Differentially expressed secondary metabolite (SM) synthetase genes and a SM gene cluster showing in planta-specific expression. Figure S8. Differentially expressed CAZY genes and secreted proteases. Figure S9. Hypoxia and oxidative stress-responsive genes are up-regulated in conidia. Figure S10. Protein domain organization and gene expression patterns of putative CFEM proteins. (PDF 1307 kb) [file 12864_2018_4934_MOESM1_ESM.pdf]

Table S1. Bioinformatic command lines used in this study

Purpose	Command lines
Reads mapping	bowtie2-build genome.fa Cf1104;<br>tophat2 -p 8 -o output -G Cf1104.gtf -g 1 -no-discordant Cf1104.index XXX.fq;<br># XXX represents a generalized RNA-seq library name
Reads counting	samtools sort XXX.bam XXX.sort;<br>samtools view XXX.sort.bam > XXX.sort.sam;<br>htseq-count -f sam -s no -r name -nonunique none XXX.sam Cf1104.gtf > XXX.table;
DEG identification in DESeq2	> library (DESeq2)<br>> countdata = read.csv (file="HTSeqTableInput.csv", header=TRUE, sep=",", row.names=1)<br># the 'HTSeqTableInput.csv' file was prepared by manually merging the htseq-count output in Excel<br><br>> Design = read.csv (file="Design.csv", header=TRUE, sep=",", row.names=1)<br># the 'Design.csv' specified the experimental design<br><br>> ddsFullCountTable = DESeqDataSetFromMatrix(countData = countdata, colData = Design, design = ~ condition)<br>> ddsFullCountTable<br>> dds = DESeq (ddsFullCountTable)<br># reads in data and calculates the normalization factors<br><br>> re = results(dds, contrast = c("condition", "XXX", "XXX"), altHypothesis="greaterAbs", lfcThreshold=3, alpha = 0.05)<br># performs DEG gene identification with indicated independent filtering (alpha = 0.05) and fold-change threshold (lfcThreshold=3) parameters<br><br>> write.csv(as.data.frame(re), file="XXX.csv")<br># write out the comparison outcome
DEG identification in edgeR	>library (edgeR)<br>> countdata = read.csv (file="HTSeqTableInput.csv", header=TRUE, sep=",", row.names=1)<br># the 'HTSeqTableInput.csv' file was prepared by manually merging the htseq-count output in Excel<br><br>> group = factor(c("CON","CON","CON","APP","APP","APP","CIH","CIH","CIH","IL","IL","IL"))<br>> keep = rowSums(cpm(countdata)>5) >= 3<br>> countkeep = countdata [keep,]<br>>d = DGEList(counts=countkeep, group=group)<br>>d$sample<br># filters away lowly-expressed genes and reads in data<br><br>>design = model.matrix (~0+group, data= d$samples)<br>>colnames(design) = levels(d$samples$group)<br>>design<br># sets up the experimental design matrix<br><br>>d = estimateDisp(d, design)<br>> fit = glmFit(d, design)<br>> tr = glmTreat(fit, contrast=c(1,-1,0,0), lfc=3)<br># performs a fold-change threshold (lfc=3) based pairwise comparison with the GLM approach<br><br>>topTags(tr)<br>> tt=topTags(tr, n=nrow(d))<br>> write.csv (tt$table, file="XXX.csv")<br># write out the comparison outcome

Table S2. qRT-PCR primers used in this study

Gene ID	Gene Name	Forward Primer	Reverse Primer
1104|07184	6PGL	CAACTACGCCCTCCTGAAGAAG	GTTGAGGTAGGTGGTGTCGATG
1104|01142	XPK1	CCCGACTTCAAGAACTACTCCAT	ACGGAAACTGGTCTGATTGAGAG
1104|11150	XPK2	CAAGGCAGGTTCAGACAAGACTC	AGAATACTGGTGTTGGGTTTGCC
1104|15728	GPDH	ACATGTGGGTGTACGAAGAAGAC	ATTTGTTGATGACGGAGGTGAGC
1104|14534	ME1	CTCTACAAGGACCAGGGCATC	GGCAGGATCTTCTCGACACC
1104|08443	ME2	TCCACAACTCCTCCAAGCTAAC	GGTGAGGTTCTTGTTGATGCG
1104|05937	FAS1	CCCAGCAGAATCTCCAAGACTT	GCATGTTGGCGATGTTGTAGTT
1104|05927	FAS2	CAAGAAGCAGATCAAGCAGTGG	GCGTACTCCTTCTCGTTGAACT
1104|07838	9FADS	GATACCACAACTTCCACCACGA	GAGCTGCTTCCACATCCAGAT
1104|15484	GPAT	TGAACTACTTCCATGCCCACAA	AATCGCCTCTCTTCTCTGGTTG
1104|05415	PDAT	AACCTGAACATCACCATCGACA	TCAAGTTGACAGTACCGTCTCC
1104|14121	2AGAT	ACTCGTTGCTGACTCTTGACAA	GATCGGACACCCATTGCTAGAA
1104|14784	DGAT1	GTGAGAGTCTGGGTGCTTACTG	CGATCAAGGGAGAGTAGACGTG
1104|10068	DGAT2	GAGTACATGGAGGGCGAGTTC	GCACCGTAGCTAAAGTACCTCA
1104|15756	Phytase	GAATGGTGCTGATGGAGATGACC	GACTTGGTCGTGGTGATGATGAG
1104|06229	PT1	AACGACTACAAACACAAGCCCAT	TTCTTGAGCATCCAGAAGAGCAC
1104|11736	PT2	GCCAGATCTACGAGTTCCACAAC	GTCGTAGTGGAGACCGTAGAGAG
1104|04742	GPDP1	GTCTACAAGCCCATCACCAACATC	TACACGACTGTAGCTTGTCCTTGA
1104|12893	GPDP2	ATTTCGAGACGTATTGGAAGGCAA	CTGGACAAACAACCGAACAAAGTC
1104|14375	GPDP3	CGACACGGAGAATTACACTATCCC	GTTTCCCACAAGTGAGTCGAGAAG
1104|14946	GPDP4	AGATCAACTCCCAGCAGACATACT	GACTTGTTGCTCGTCAGGTTCTTA
1104|15920	GPDP5	GATGGTCTCAACATGCCGAAGAAG	ATTTCTGTGGTTGTGAGGTGGTAG
1104|04754	qutD	CAGTTGCACCATTGGCTACGA	CTGTAGGACGCGAACTGGAAC
1104|04738	qutB	CCACCGACATTTACCTCGTCAA	GGTACAGGTTGTCTCCATAGCC
1104|04714	qutE	TCAGACACCACAGCTACTTGAG	CTTCTGCTTAATCCTCATGTGCTT
1104|04744	qutC	ATGCCTTCACGCCTGGGTAT	TTTGCTGCCATGTCCATCTTGT
1104|03859	aromA	AAAGCCAATTTCTCAGCCCTAC	CTCGGAATGTGATAGGTGTTGG
1104|06927	CHS	CTCTCCCTCCTCAACACCATC	TGCAGTCAATCATCTTCTCGTT
1104|03845	Tubulin a	CCACTTCCCTTTGGTCGCTTAC	CATGGTCATCTCCTGGACAGAGT

Table S3. Summary statistics of the reads mapping outcomes for individual RNA-Seq libraries

Library	Raw Paired Reads	Clean Paired Reads	Clean Base (G)	Aligned Pairs	Multiple Hit Pairs	Discordant Pairs	Concordant Unique Pairs
CON-1	5,296,972	5,228,005	1.57	3,942,679	9,348	4,485	3,928,846 (75.2%)*
CON-2	6,419,268	6,308,459	1.89	4,902,911	11,855	5,020	4,886,036 (77.5%)
CON-3	7,497,064	7,391,219	2.22	5,471,634	13,844	8,553	5,449,237 (73.7%)
APP-1	4,430,508	4,325,588	1.30	3,339,044	8,691	3,253	3,327,100 (76.9%)
APP-2	5,420,623	5,304,240	1.59	3,907,507	9,235	4,248	3,894,024 (73.4%)
APP-3	5,139,597	5,024,377	1.51	3,835,006	9,330	9,851	3,815,825 (75.9%)
CIH-1	4,345,583	4,116,515	1.23	3,169,234	13,756	5,784	3,149,694 (76.5%)
CIH-2	4,728,212	4,477,571	1.34	3,271,745	14,490	6,402	3,250,853 (72.6%)
CIH-3	6,182,049	5,891,733	1.77	4,268,383	25,591	6,827	4,235,965 (71.9%)
IL-1	30,846,670	30,623,705	9.19	164,987	1,342	100	163,545 (0.53%)
IL-2	33,017,109	32,636,921	9.79	424,027	3,136	145	420,746 (1.29%)
IL-3	35,535,538	35,189,616	10.56	219,594	2,079	73	217,442 (0.62%)

*concordant alignment rate

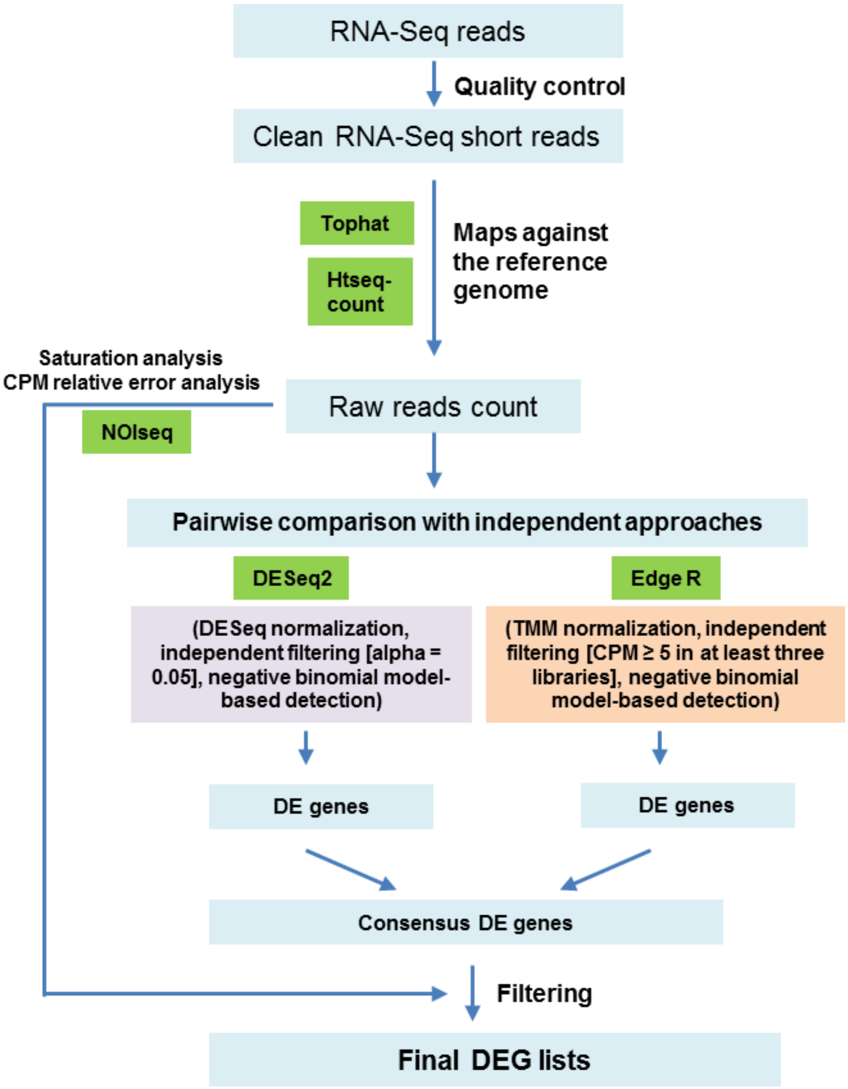

Figure S1. Steps of identifying differentially expressed genes.

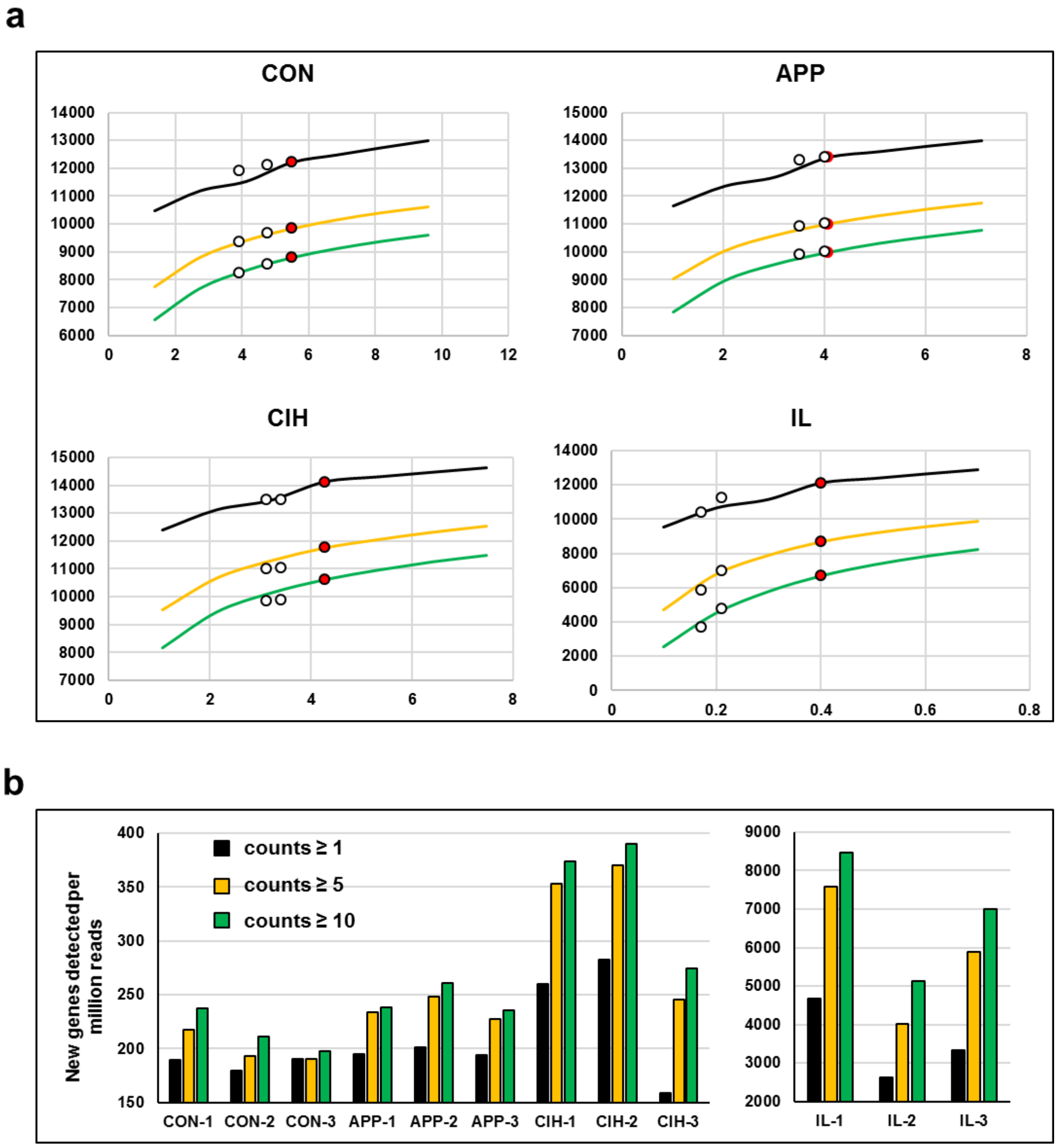

Figure S2. Saturation analysis of HTSeq reads count dataset. **a**. Saturation curves. Each line represents the numbers of detected genes (y-axis) at indicated sequencing depths (x-axis, million reads). Lines were simulations based on CON3, APP2, CIH3, and IL2 libraries (red dots), additional RNA-seq libraries were plotted as filled circles. Different line colors represent simulations with different counts cutoffs (black, counts ≥ 1; yellow, counts ≥ 5; green, counts ≥ 10). **b**. Expected number of new genes detected per million of new sequenced reads at the sequencing depth of each RNA-seq library. The calculations were based on the formula 'expected new genes per million reads = (expected genes detected at the highest simulation depth – genes detected at the library depth)/(the highest simulation depth - the library depth).

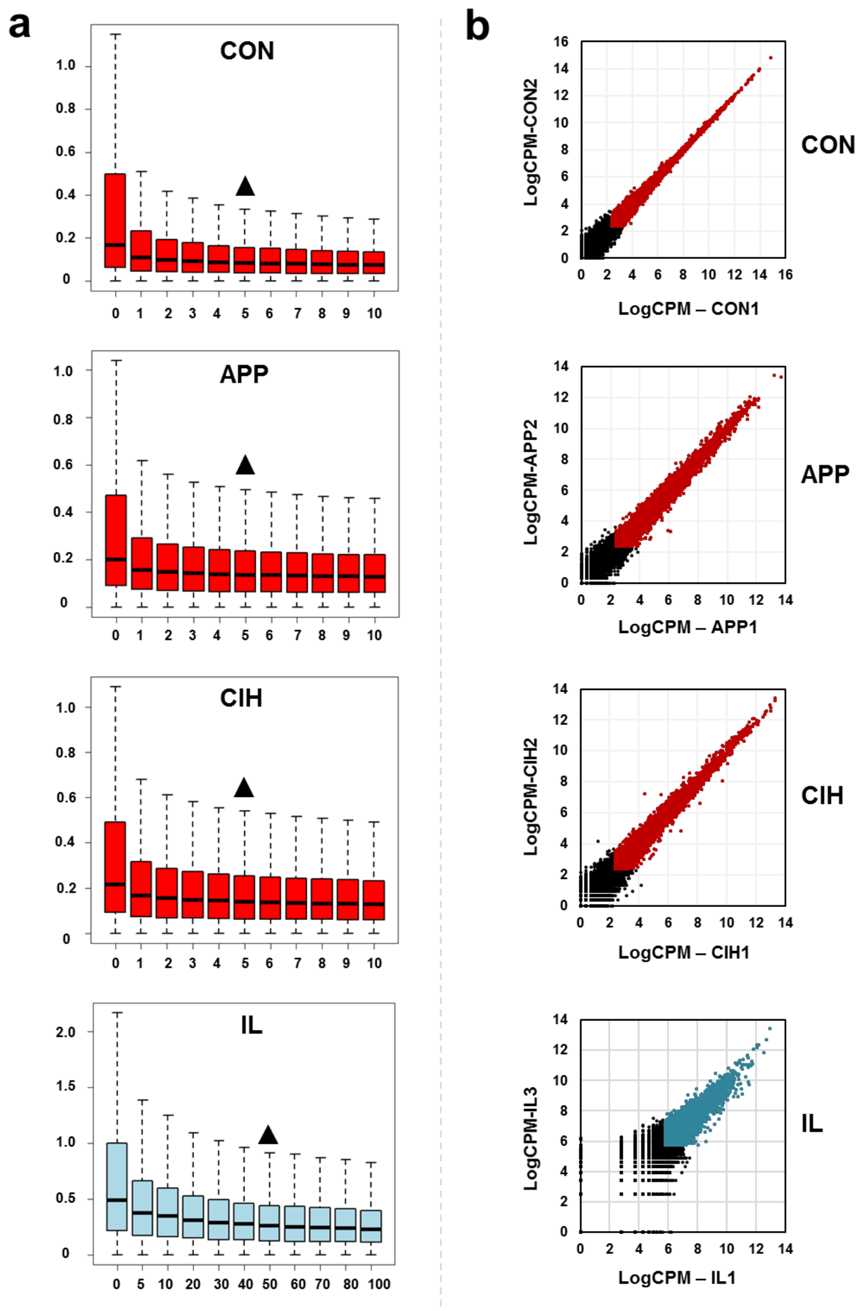

Figure S3. Defining the criterion for filtering lowly expressed genes. **a**. Boxplots showing the distributions of relative CPM errors (y-axis) after removing lowly-expressed genes at different CPM filtering thresholds (x-axis). For each gene, the relative CPM error was calculated as the relative difference between the average CPM in one replicate (chosen arbitrarily) and the average CPM calculated from the rest two replicates. Black triangle indicates the chosen CPM threshold. **b**. Correlation plot of log2 transformed CPM values; color dots represent genes with CPM values higher than the chosen thresholds.

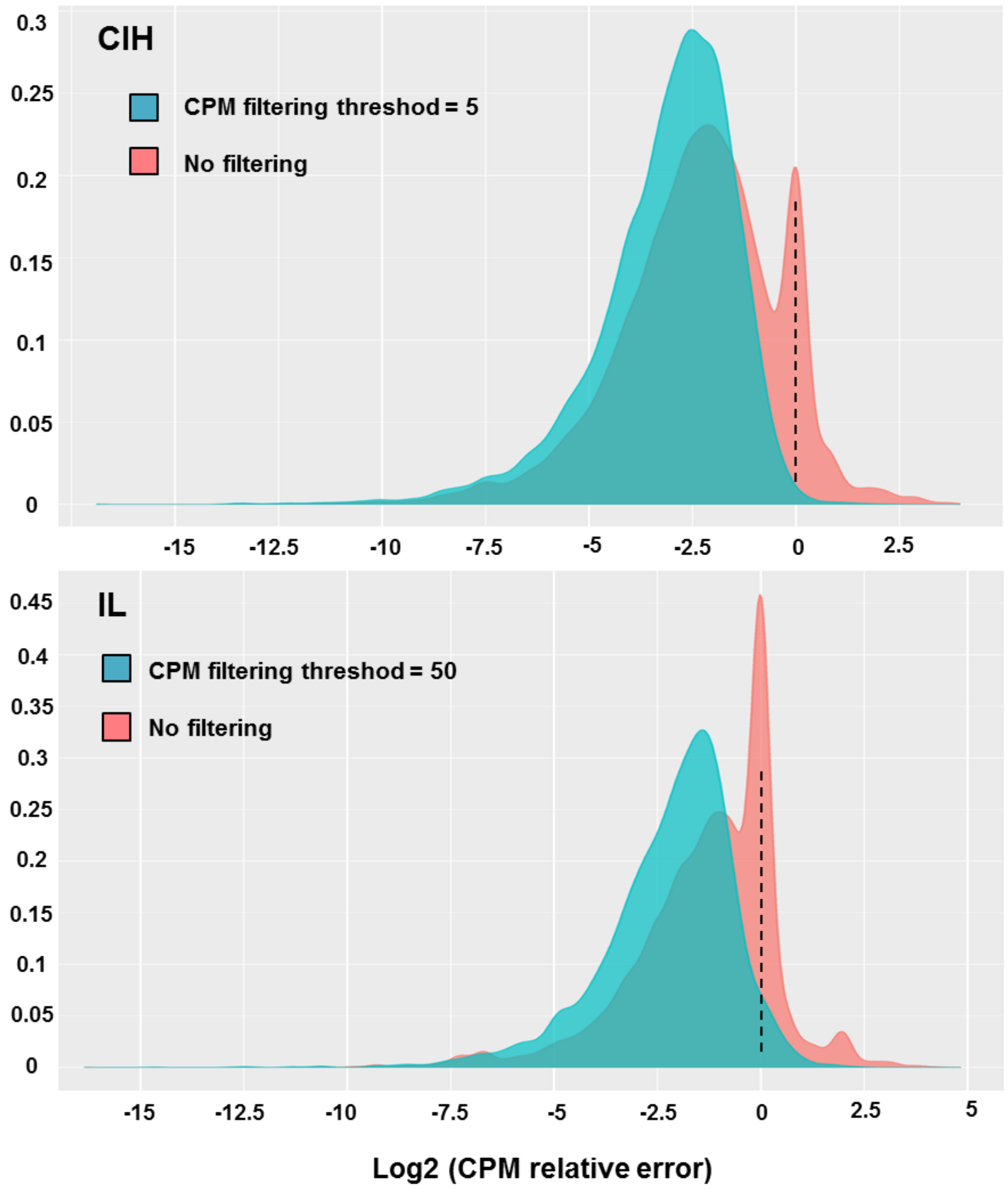

Figure S4. Density plot showing the effect of gene filtering on the distribution of relative CPM errors for the CIH (top) and IL (bottom) conditions. The x-axis represents Log2 (CPM relative error) values, for which zero (the dashed vertical black line) corresponds to two-fold expressional (CPM) difference between the chosen replicate and the average CPM of the rest two replicates.

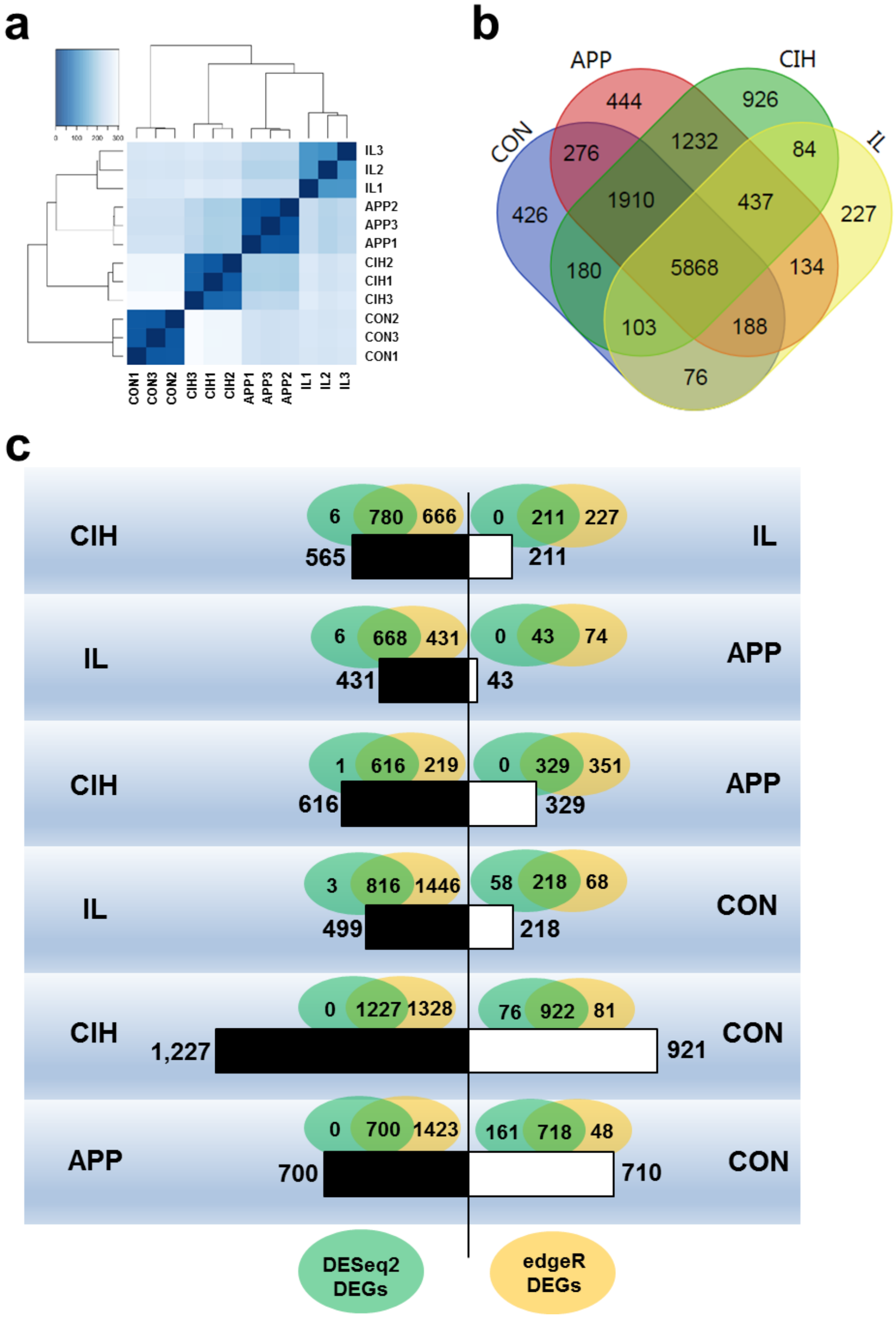

Figure S5. Identification of differentially expressed genes (DEGs). **a**. Heatmap showing the relationships of the 12 RNA-seq datasets. **b**. Venn diagram showing genes detected to be expressed (CPM ≥ 1 in all three libraries for CON, APP, and CIH; CPM ≥ 10 in all three libraries for IL). **c**. Number of genes showing significant expressional differences among pairwise comparisons. DEGs being co-supported by DESeq2 and edgeR (indicated by the Venn diagram) were further filtered with CPM thresholds (average CPM ≥ 5 for CON, APP, and CIH; average CPM ≥ 50 for IL) to give the final outcome (numbers next to horizontal bars).

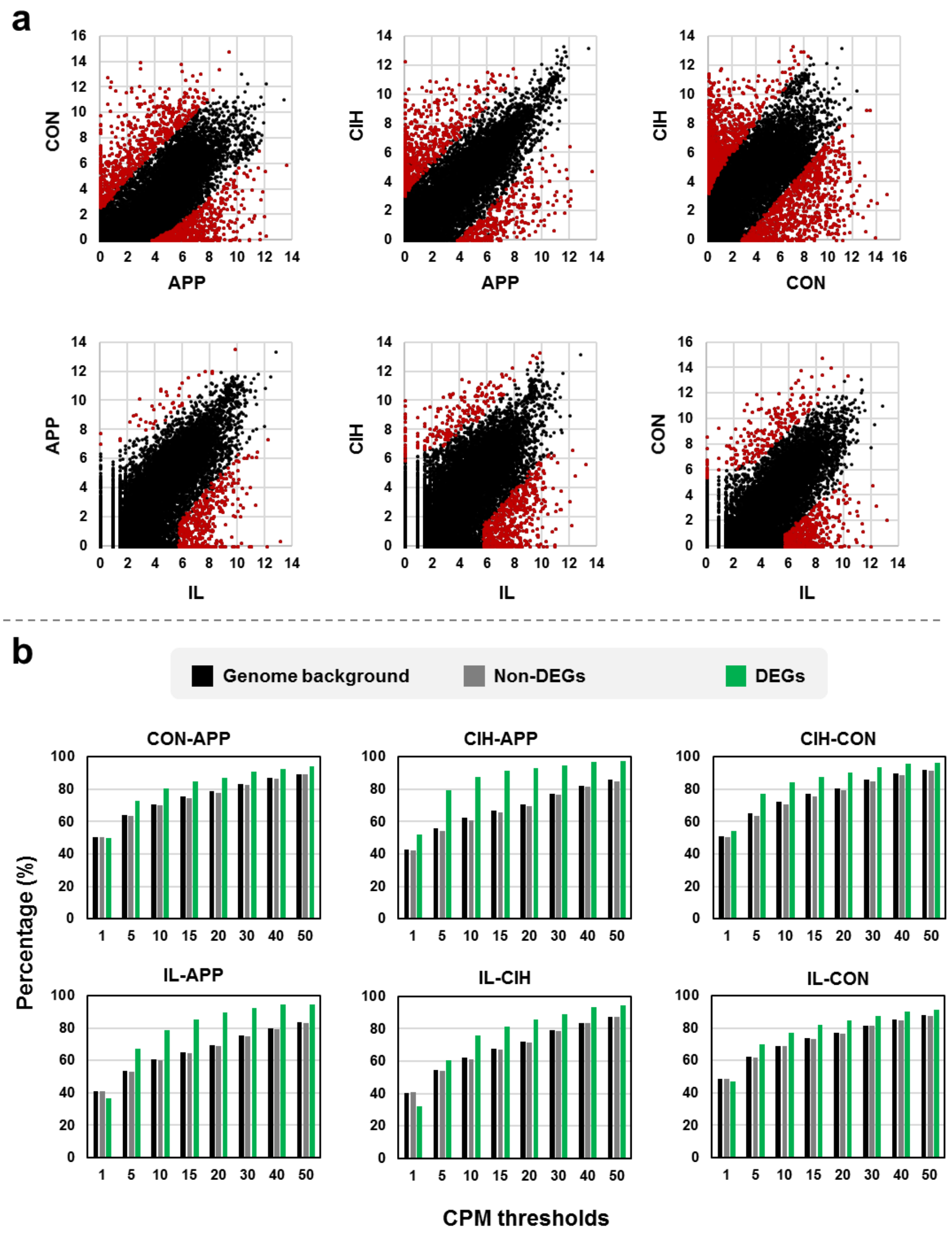

Figure S6. CPM distribution patterns of identified DEGs. **a**. Average CPM values were log2 transformed to generate the correlation plots, in which DEGs are shown as red dots and non-DEGs are shown as black dots, x- and y-axis values represent log transformed average CPMs. **b**. The proportion of genes with CPM values less than a given threshold in at least one of the two compared samples. For each pairwise comparison and for each CPM threshold (1, 5, 10, 15, 20, 30, 40, 50), proportions for three categories of genes were calculated (bars in different colors). Genome background means all predicted gene models being considered.

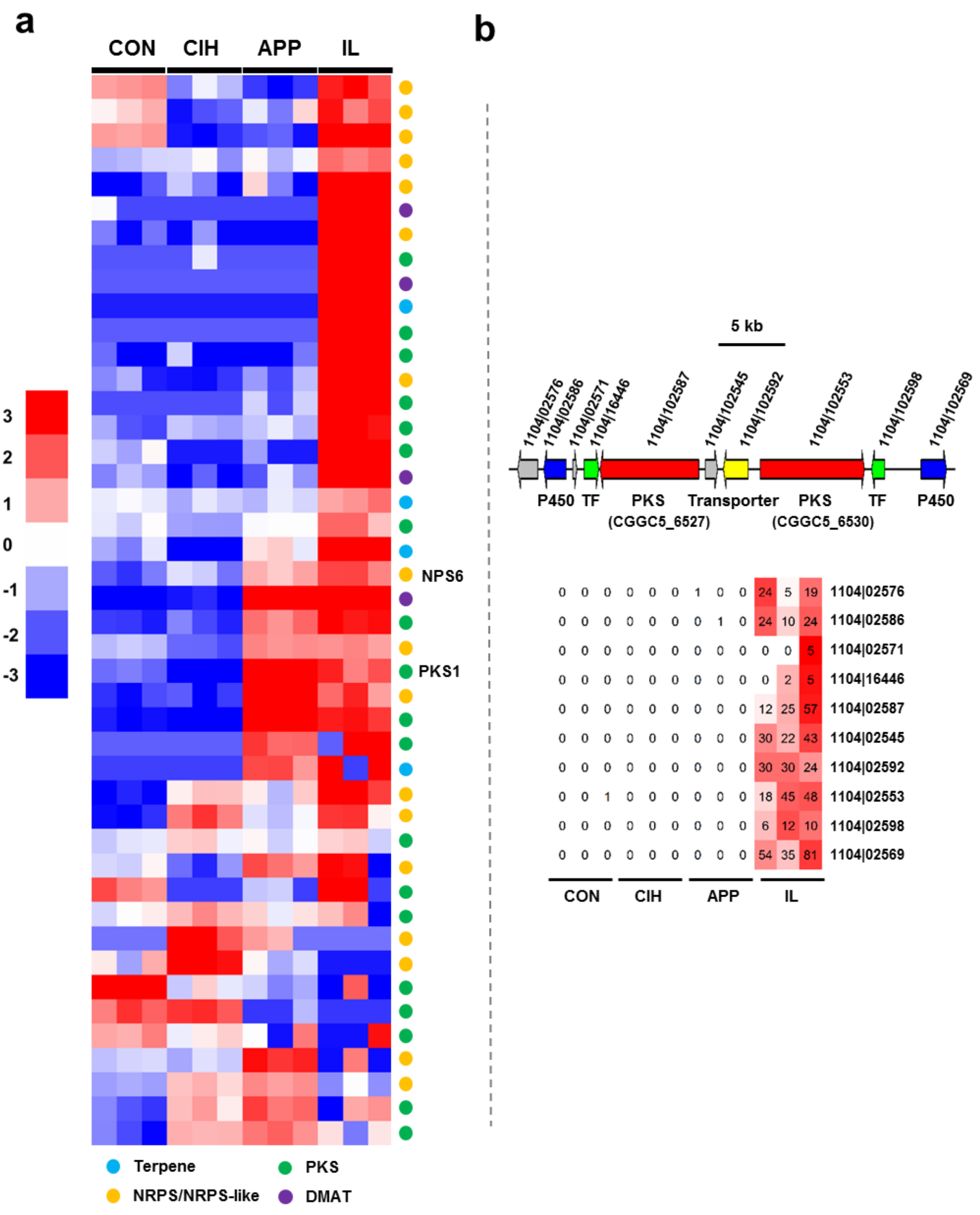

Figure S7. Differentially expressed secondary metabolite (SM) synthetase genes (**a**) and a SM gene cluster showing *in planta*-specific expression (**b**). Number indicates normalized read count value.

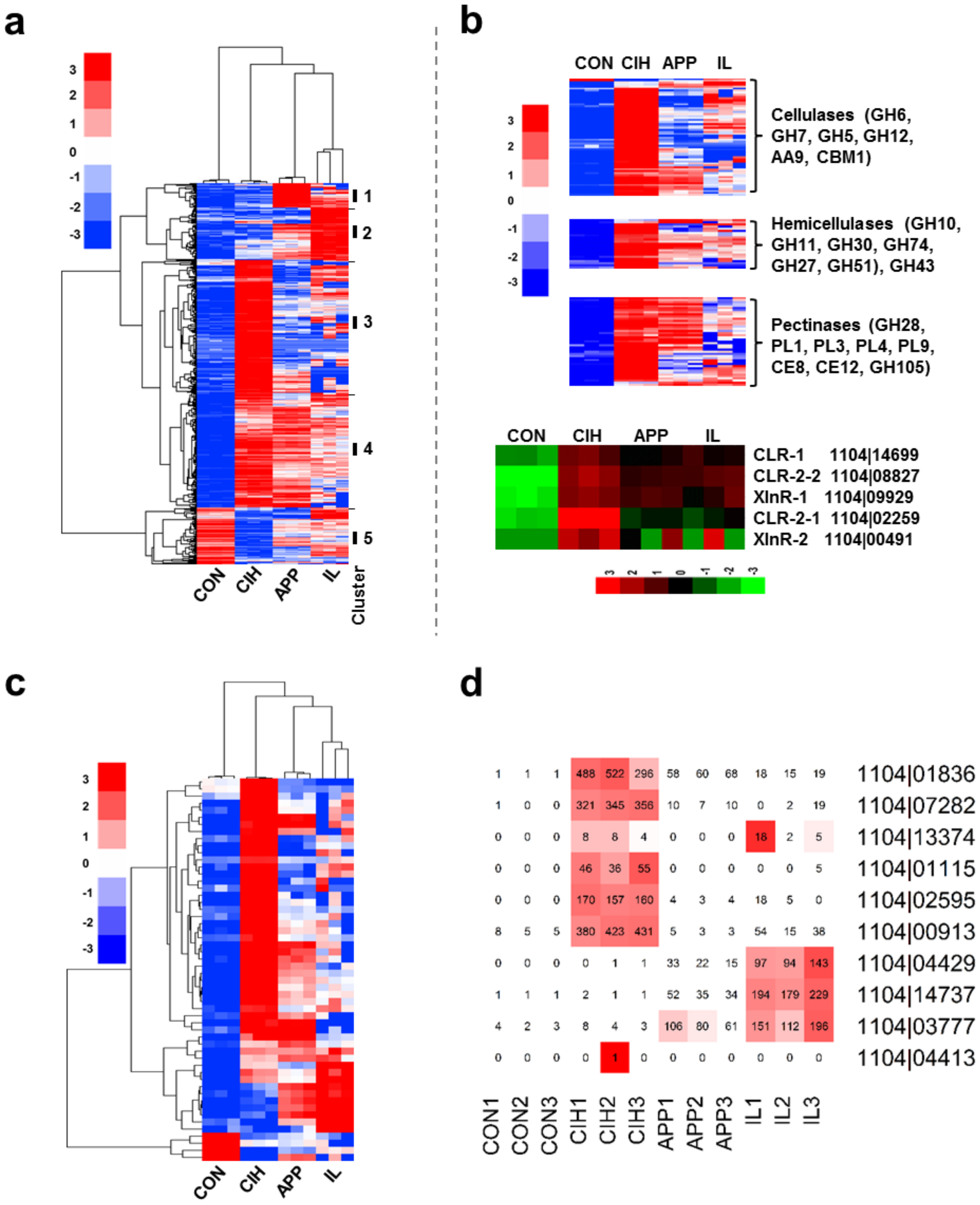

Figure S8. Differentially expressed CAZY genes and secreted proteases. **a**. Hierarchical clustering of the 422 differentially expressed CAZY genes. **b**. Expressional profiles of the 126 differentially expressed PCWDEs (top) and transcriptional regulators (bottom). **c**. Hierarchical clustering of the 54 differentially expressed secretory proteases. **d**. Normalized HT-seq count values for secretory proteases belonging to the M14A and S53A families.

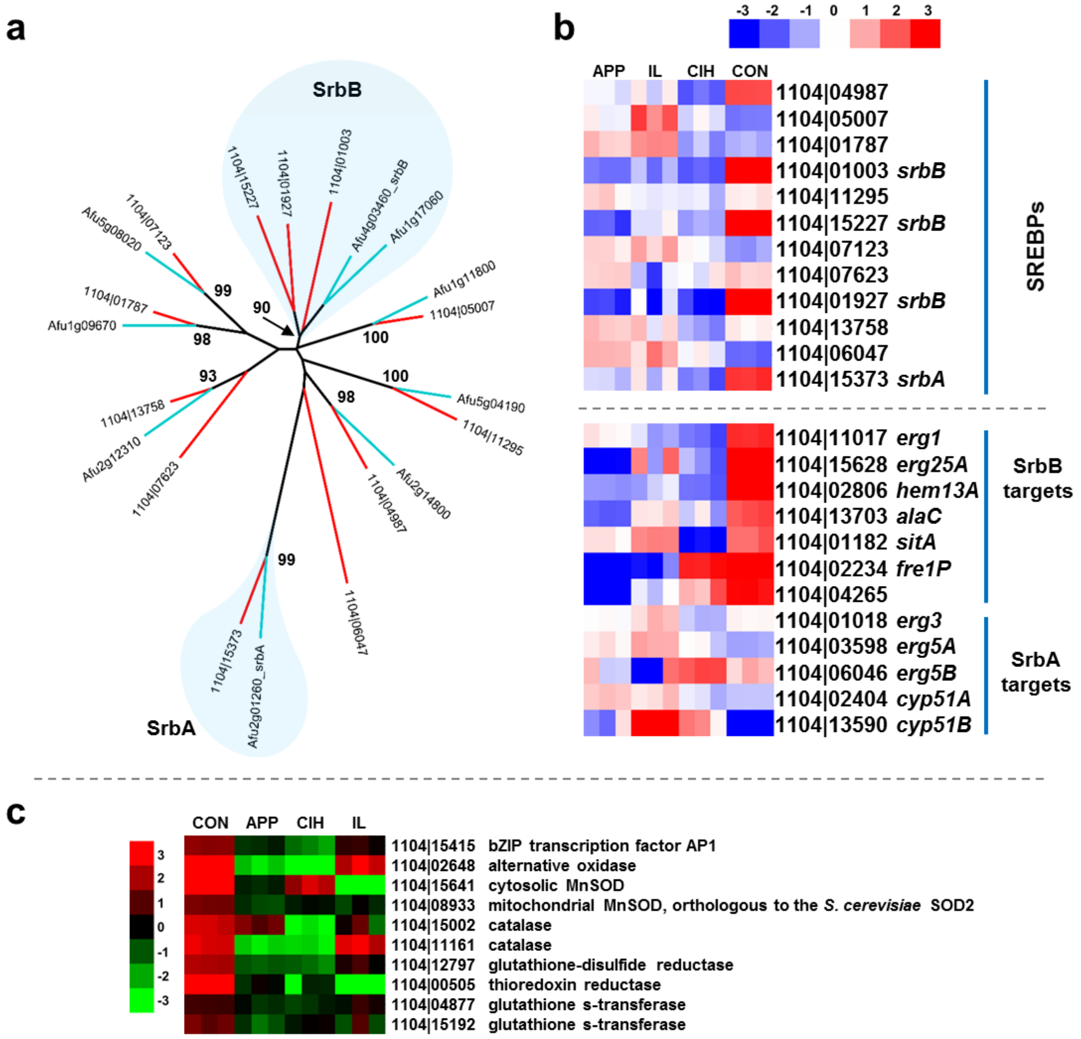

Figure S9. Hypoxia and oxidative stress-responsive genes are up-regulated in conidia. **a**. Phylogenetic relationship of transcription factors of the sterol regulatory element binding protein (SREBP) class from *C. fructicola* and *Aspergillus fumigatus*. The 1104-7 genome contained one *SrbA* and three *SrbB* homologues. **b**. Heatmap expression patterns of SREBPs and downstream targets of SrbA and SrbB; log2 ratios of CPMs are plotted. **c**. Heatmap expression patterns of oxidative stress-responsive genes.

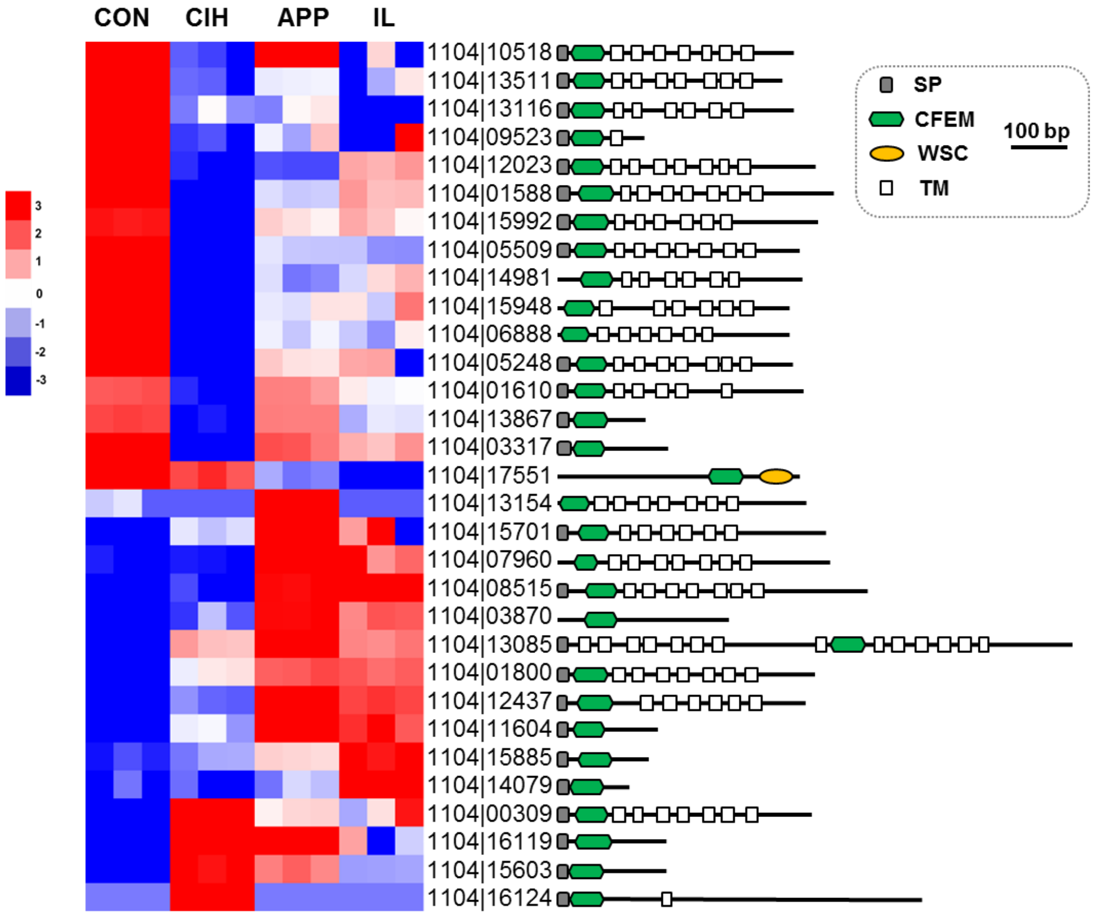

Figure S10. Protein domain organization and gene expression patterns of putative CFEM proteins.
